# N-Unet: An Efficient Multi-Task Model for Precise Classification and Segmentation of Breast Ultrasound Images

**DOI:** 10.3390/jimaging12050194

**Published:** 2026-04-30

**Authors:** Yafeng Yang, Zhengwei Zhu

**Affiliations:** Microelectronics and Control Engineering, Changzhou University, Changzhou 213011, China; 14461228@smail.cczu.edu.cn

**Keywords:** multi-task learning, deep learning, image segmentation, medical image analysis, breast ultrasound diagnosis

## Abstract

Deep learning has substantially advanced the automated classification and segmentation of breast ultrasound images. However, many existing methods do not fully exploit task correlations, which weakens information exchange and limits the delineation of fine structures. In addition, commonly used loss functions often fail to balance classification and segmentation objectives effectively. To address these issues, we propose N-Unet, a multi-task learning framework that combines adaptive optimization with feature-enhancement modules. Specifically, the Adaptive Multi-Task Loss (AMTL) dynamically balances the two task objectives to promote stable joint learning. The Adaptive Feature Fusion (AFF) and Cross-Level Attention Enhancement (CLAE) modules improve feature representation through multi-scale integration and semantic refinement. The Conditional Segmentation Boosting (CSB) module further refines segmentation outputs according to the classification result, improving inference-stage consistency. Together, these components form a unified multi-task framework with a shared encoder, a segmentation branch, and an integrated classification branch whose output further supports segmentation-consistency refinement. Experiments on the BUSI and BUS-UCLM datasets demonstrate the superiority of N-Unet. The model achieves classification accuracies of 96.54% on BUSI and 95.83% on BUS-UCLM, with corresponding Dice scores of 80.70% and 92.16%. It reaches this performance with only 8.95 M parameters and 14.74 GFLOPs, showing a favorable performance-efficiency trade-off. These results confirm the effectiveness of N-Unet and its robustness across the two BUS datasets studied here, supporting its potential for practical breast nodule assessment, while broader external generalization remains to be validated.

## 1. Introduction

Cancer remains a major global health burden, and accurate, timely diagnosis is essential for early intervention and improved survival. Among cancers affecting women, breast cancer is of particular concern [1]. According to the global cancer statistics for 2020 released by the International Agency for Research on Cancer (IARC), 2.26 million new breast cancer cases were reported worldwide, more than for any other cancer in women [2]. In addition, breast cancer caused 685,000 deaths worldwide and remains the leading cause of cancer-related death among women. Survival strongly depends on the stage at diagnosis: localized disease has a 99% survival rate, regional disease 86%, and distant metastasis only 30% [3]. These figures highlight the importance of early detection in improving patient outcomes.

To detect breast cancer at an early stage, clinicians often rely on BUS imaging because it is non-invasive, radiation-free, cost-effective, and widely available. However, ultrasound image quality depends heavily on operator expertise, and inexperienced examiners may produce suboptimal images that are more difficult to interpret. Breast tumor identification and classification are further complicated by the joint influence of lesion characteristics, surrounding tissue, and acquisition conditions. In practice, BUS images often suffer from low contrast, speckle noise, and large variations in tumor size. In addition, breast lesions may exhibit iso-intensity, in which different tissue types have similar visual appearances, or hypointensity, in which lesions appear as darker regions with reduced signal. Patient-specific anatomical variation and inconsistent image quality further increase diagnostic difficulty. As a result, manual BUS assessment remains time-consuming, labor-intensive, and susceptible to misdiagnosis.

MTL has strong potential for BUS nodule classification and segmentation. In conventional single-task settings, the two tasks are usually handled independently and often require separate models [4,5]. By contrast, MTL trains a single model on multiple related tasks and allows them to share information. This setup improves efficiency and can also enhance the performance of each task. In BUS analysis, classification and segmentation are inherently related [6]: accurate segmentation can support classification, while reliable classification cues can assist segmentation. A well-designed multi-task model therefore has the potential to improve both tasks simultaneously.

Given these characteristics, many MTL studies have shown strong promise in this domain [6,7,8,9]. By jointly performing classification and segmentation, MTL can improve discrimination while partially alleviating the data imbalance caused by the unequal numbers of benign and malignant nodules. Moreover, MTL can automatically learn which features or regions are important for each task, providing a more flexible and accurate approach than single-task models.

A representative MTL framework is based on the Unet model, which extracts image features through the encoder and separately performs classification and segmentation through different decoders. To better capture the characteristics of benign and malignant nodules, attention mechanisms or other advanced components can be incorporated into this framework. Therefore, jointly training the two tasks within a single network, while promoting feature sharing between breast tumor classification and segmentation, constitutes a promising direction for further research.

Deep learning for medical image analysis still faces several major challenges. First, the growing demand for MTL exposes the limitations of traditional single-task models, which are designed for either segmentation or classification rather than joint image understanding. Second, effective feature fusion and information flow remain difficult in deep architectures, especially when features from multiple levels and scales must be integrated efficiently. Third, although existing attention mechanisms are effective in specific settings, each has its own limitations. For example, CBAM [10] and Attention Gate [11] offer different advantages, yet research on combining them effectively for BUS analysis remains limited. Fourth, although previous studies have explored loss optimization for MTL [12,13], it remains challenging to design a loss function that simultaneously addresses class imbalance and multi-task optimization. Finally, reliability and robustness during inference are often less studied than performance during training. To address these issues, we propose N-Unet, which incorporates several new modules and a tailored loss design to improve both accuracy and practical reliability.

Extensive experiments on publicly available BUSI data show that the proposed model provides clear improvements in both tasks compared with existing methods. Our main contributions are summarized as follows:

1. We introduce an innovative multi-task loss function, AMTL, which effectively balances optimization across different tasks and improves overall performance.
2. We propose a medical image analysis model called N-Unet, which includes two novel modules: the Adaptive Feature Fusion (AFF) module and the Cross-Level Attention Enhancement (CLAE) module. By combining attention mechanisms with feature fusion, these modules improve feature extraction and information flow.
3. We further propose an inference-stage optimization strategy, Conditional Segmentation Boosting (CSB). This module uses classification results to assist and refine segmentation, thereby improving model reliability and robustness in practical applications.
4. We evaluate N-Unet on the BUSI and BUS-UCLM datasets, showing strong classification and segmentation performance with only 8.95 M parameters and 14.74 GFLOPs.

These experimental results further support the effectiveness and practical applicability of the proposed model.

## 2. Related Work

### 2.1. Classification

In breast ultrasound image analysis, deep learning has become an important tool for accurate diagnosis. Progress in these methods has substantially improved classification performance and provided strong technical support for the early detection and treatment of breast cancer. For example, the authors of [14] proposed an ensemble of pretrained VGG19 [15] and ResNet152 [16] models for BUS classification, achieving an accuracy of 90%. In addition, ref. [17] proposed an image-fusion approach and trained an ensemble of CNNs using different image representations. Their method achieved 91% accuracy on a private dataset and more than 94% accuracy on the publicly available BUSI dataset.

Although most existing studies on breast ultrasound analysis focus on distinguishing benign from malignant nodules, identifying whether a lesion is present at all is equally important in real clinical diagnosis. Timely detection of abnormalities has a direct impact on treatment effectiveness and clinical response. This study addresses that need by applying advanced deep learning techniques to classify breast ultrasound images into lesion-present and lesion-free categories, thereby providing more comprehensive support for clinical diagnosis.

Another challenge in lesion detection is the inherent imbalance of the dataset. For example, in the BUSI dataset, images containing nodules substantially outnumber normal images without nodules. This imbalance can cause the classification model to overfit the majority class and perform poorly on the minority class. To address this issue, we propose an improved loss function that enhances classification performance across all categories while paying particular attention to the detection of non-lesion samples.

### 2.2. Segmentation

To assist radiologists in interpreting BUS images and improving diagnostic accuracy, computer-aided diagnosis (CAD) systems have been widely developed, with image segmentation serving as a critical component [18,19]. Deep learning, particularly convolutional neural networks (CNNs), has shown significant advantages over traditional segmentation techniques in handling the variability and complexity of breast tumors in terms of shape, texture, and boundary definition [20,21].

Among deep learning-based approaches, Unet and its derivatives remain the foundation of most modern segmentation models [22]. Variants such as ANUnet [23], which employs attention gating to highlight salient regions, and MultiResUNet [24], which introduces multi-resolution analysis paths, have demonstrated improved performance in delineating complex and blurry tumor boundaries. These architectures focus on enhancing spatial and semantic feature representation to achieve more accurate segmentation in medical images.

Furthermore, the integration of Transformer-based modules has recently gained traction in medical image segmentation due to their strong ability to capture long-range dependencies and global context. Models such as UNETR [25] and SwinUnet [26] leverage transformer-based representations within Unet-like structures to improve segmentation accuracy across challenging image analysis tasks. These methods highlight the potential of hybrid architectures that combine the locality of CNNs with the global modeling capacity of Transformers.

In this study, we build upon these advancements by integrating attention mechanisms and task-specific guidance into a multi-task learning framework, aiming to simultaneously optimize segmentation and classification performance in BUS images.

### 2.3. Hybrid Tasks

Recent advances in MTL have demonstrated significant advantages in breast ultrasound imaging, particularly in jointly optimizing classification and segmentation tasks. The fundamental principle of MTL lies in leveraging shared representations to enhance feature learning across interrelated tasks, thereby improving overall model generalization.

Several recent studies have introduced innovative MTL frameworks for breast ultrasound analysis. Dai et al. [27] proposed MTF-Unet, a multi-scale, multi-task model that employs a uniform convolutional kernel size with varying depths to extract multi-scale, multi-level features. It further integrates an adaptive fusion block to improve information exchange across feature levels, enhancing segmentation accuracy through auxiliary classification guidance. Similarly, Aumente et al. [28] introduced BTS-Unet, an end-to-end multi-task architecture designed to exploit the inherent correlation between classification and segmentation. By incorporating a widely used breast cancer ultrasound dataset, BTS-Unet demonstrated the effectiveness of a balanced segmentation-classification learning scheme. On the other hand, He et al. [29] developed ACSNet, which refines MTL by introducing deformable spatial attention modules to improve segmentation accuracy while simultaneously optimizing classification through feature enhancement. ACSNet outperformed mainstream MTL frameworks by achieving superior segmentation and classification performance on breast ultrasound datasets.

Beyond CNN-based frameworks, recent years have also witnessed growing interest in Transformer-based architectures for multi-task medical image analysis. Yang et al. [30] developed a Transformer-based multi-task model for simultaneous T-stage identification and gross tumor volume segmentation in nasopharyngeal carcinoma, demonstrating that joint Transformer-based learning yields mutual performance gains across both tasks. Xu et al. [31] proposed PointFormer, which decouples nuclei segmentation and classification into a keypoint-guided tri-decoder Transformer structure with an inter-branch attention guidance strategy, achieving state-of-the-art performance in multi-tissue histopathology. Nath et al. [32] presented MTMedFormer, which employs a shared Transformer encoder and task-specific decoders for simultaneous diagnostic tasks across mammography and chest imaging, and further integrates federated learning to address data privacy constraints. In the breast ultrasound domain, Tagnamas et al. [33] proposed a hybrid CNN-Transformer multi-task framework with a dual-encoder design based on EfficientNetV2 and an adapted Vision Transformer, integrated via a Channel Attention Fusion module, for simultaneous tumor segmentation and classification. These advances collectively reflect the growing consensus that Transformer-based multi-task architectures hold strong potential for complex medical imaging tasks. However, this representational power generally comes at the cost of substantially higher parameter counts and computational requirements compared with CNN-based architectures, a trade-off that warrants consideration in resource-constrained clinical settings.

In MTL, the design of the loss function is particularly important because it directly affects the importance assigned to different tasks and the efficiency of learning. For example, Kendall et al. [12] emphasized the importance of task weights in MTL and argued that manual tuning is complex and costly. To address this issue, they proposed a principled method that automatically weighs task losses by modeling the homoscedastic uncertainty of each task. This approach avoids manual tuning and allows the system to adapt to task importance, leading to improved performance.

Chennupati et al. [34] further proposed using the geometric mean of task losses instead of weighted averaging. Compared with the conventional arithmetic mean, the geometric mean better balances differences in convergence speed across tasks, which is especially important when task importance varies substantially. Their experiments demonstrated the superiority of this loss over existing multi-task solutions on a wide range of datasets. Focal Loss, proposed by Lin et al. [35] for object detection, is also highly effective for imbalanced datasets. Its main idea is to modify cross-entropy loss so that the model focuses more on difficult samples and less on easy ones. In this study, we combine Focal Loss with other loss functions to build a more balanced and accurate MTL model.

Despite these advances, several important limitations remain in existing multi-task approaches for breast ultrasound analysis. First, most methods, whether CNN-based or hybrid Transformer-based, rely on fixed or manually tuned task weights that do not adapt to changing optimization dynamics during training. Second, cross-level feature fusion is commonly built on only one type of attention mechanism, without jointly exploiting channel attention and spatial attention gating for richer semantic alignment across encoder-decoder levels. Third, few frameworks explicitly use classification confidence to refine segmentation outputs during inference, even though such guidance is important for clinical reliability. Fourth, Transformer-based methods, despite their strong representational capacity, usually introduce high computational and parameter costs, which can limit their practicality in small clinical datasets. In response to these limitations, N-Unet combines an Adaptive Multi-Task Loss (AMTL) with Adaptive Feature Fusion (AFF), Cross-Level Attention Enhancement (CLAE), and Conditional Segmentation Boosting (CSB). Together, these components strengthen task interaction, improve inference-stage consistency, and retain a lightweight footprint of only 8.95 M parameters and 14.74 GFLOPs, making the model more practical for data-limited clinical settings.

By incorporating these latest developments in MTL, our study pushes the boundary of breast ultrasound image analysis, demonstrating that carefully designed MTL architectures can significantly enhance both classification and segmentation accuracy.

## 3. Method

### 3.1. Overall Architecture of N-Unet

Medical image segmentation is pivotal in automated diagnosis and treatment planning, necessitating robust feature extraction and precise boundary delineation. To address these challenges, we introduce N-Unet, a novel multitask learning framework designed to enhance the classification and segmentation performance of breast ultrasound images.

N-Unet extends the classical Unet backbone by incorporating several key innovations, namely the AFF module, the CLAE module, and a classification-driven Conditional Segmentation Boosting (CSB) mechanism. The model follows a hybrid multi-scale encoding strategy, effectively capturing hierarchical spatial features while maintaining inter-task coherence.

**Figure 1:**
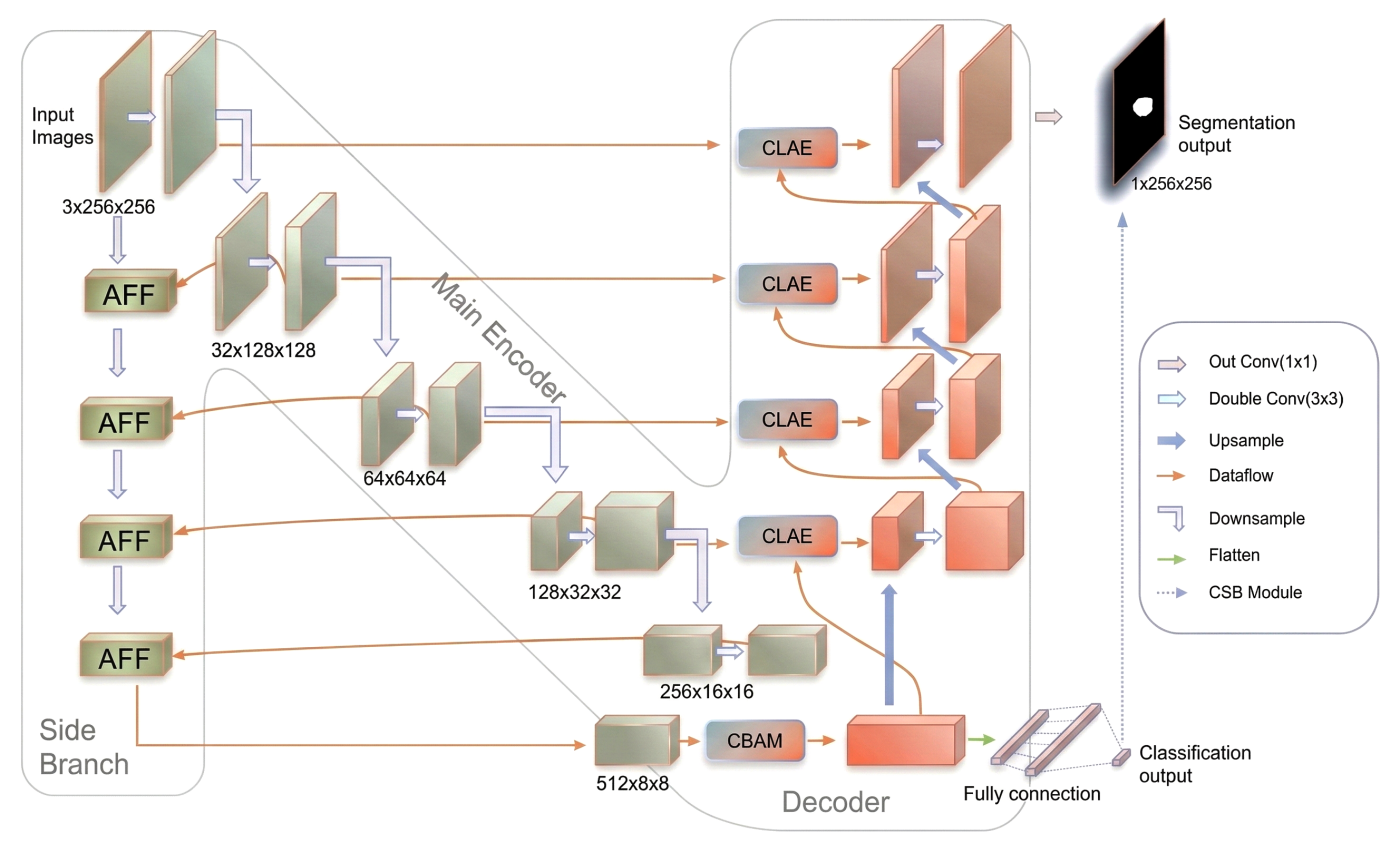
Overall architecture of N-Unet. The model consists of an enhanced encoder-decoder structure incorporating Adaptive Feature Fusion (AFF) modules, Cross-Level Attention Enhancement (CLAE) modules, and a CBAM-based classification refinement mechanism. The left section represents the feature extraction and downsampling stages, while the right section highlights the multi-scale feature integration and segmentation pathways. In this schematic, the encoder is shown by the green-toned left pathway together with the diagonal main-encoder branch, whereas the decoder is shown by the red/orange-toned right reconstruction pathway. At the 256×16×16 level, the right-hand feature block already has a visible outgoing connection to the final AFF module before the network continues to the 512×8×8 bottleneck. The pale-blue hollow arrows denote the Double Conv (3×3) operation; in the rendered figure, some of these arrows appear white-filled because of the light interior shading, but they represent the same symbol rather than a separate operation.

As illustrated in Figure 1, the architecture comprises three main components: feature extraction and downsampling, cross-level feature refinement, and classification-assisted segmentation. Within this unified framework, the segmentation branch performs decoder-side reconstruction, whereas the classification branch functions as an integrated image-level prediction head operating on the same shared encoder representation. The two branches are optimized jointly through AMTL, and the classification output is further reused by CSB during inference to refine segmentation consistency. The left side of the network forms the encoder pathway, corresponding to the green-toned AFF-enhanced branch together with the diagonal main-encoder path in Figure 1. Each level is enhanced by an AFF module, which adaptively fuses multi-scale features to strengthen contextual representation. Unlike a standard Unet encoder, this design promotes richer information exchange during downsampling. The right side of the network forms the decoder, corresponding to the red/orange-toned reconstruction branch, where CLAE modules integrate multi-resolution features through combined CBAM and attention-gate operations to improve semantic consistency during reconstruction. Figure 1 adopts a compact topology-level layout, in which the 256×16×16 stage continues to the final AFF module and then to the 512×8×8 bottleneck before entering the decoder. Accordingly, the apparent “N”-shaped arrangement reflects the compact organization of the side branch, main encoder, and decoder rather than omitted effective connections. To further improve segmentation reliability, the classification-driven CSB module refines the final segmentation output according to the classification result, reducing semantically inconsistent predictions.

Through these enhancements, N-Unet improves segmentation accuracy while benefiting from classification-guided refinement. The coordinated use of adaptive attention and feature fusion modules enables the model to generalize more effectively across variable imaging conditions, providing a more accurate and robust solution for breast ultrasound analysis.

The following sections describe the design principles, operating mechanisms, and implementation details of the main N-Unet components. The AFF subsection explains how contextual relevance is strengthened during downsampling. The CLAE subsection describes how channel attention, spatial attention, and attention gating are combined to refine upsampled features. The CSB subsection explains how classification results guide segmentation refinement. Finally, the AMTL subsection introduces a tailored multi-task loss function for balancing classification and segmentation.

### 3.2. Adaptive Feature Fusion Mechanism (AFF)

**Figure 2:**
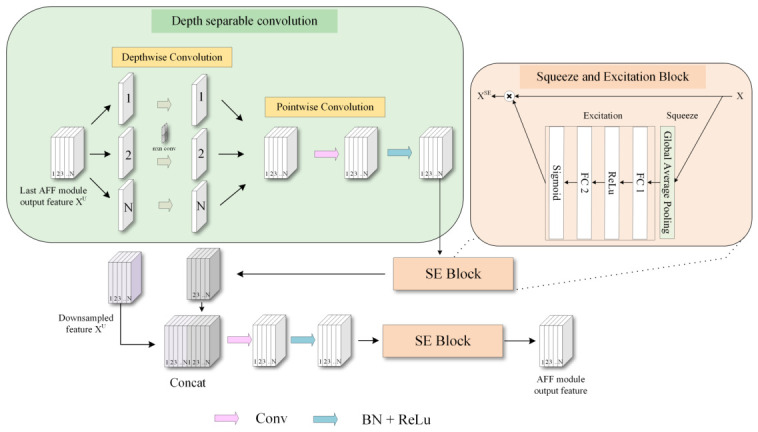
Adaptive Feature Fusion Module (AFF Module). In this schematic, the black arrows indicate the main feature-flow direction, the pale green arrows in the depthwise-convolution panel denote channel-wise filtering by the per-channel n×n convolution, the dotted lines indicate the enlarged SE-block schematic, and ⊗ denotes channel-wise reweighting of the feature map by the SE attention weights.

The AFF module is a core component of the N-Unet encoder. It integrates multi-scale contextual information through a parallel side branch, enriching feature representations during downsampling and improving the model’s perception of both global and local patterns. As shown in Figure 2, the AFF module contains the following main stages:

1. Depth-wise separable convolution (DS) [36]: DS is an efficient alternative to standard convolution. It decomposes the operation into two consecutive steps: depth-wise convolution and point-wise convolution.
  In the depth-wise stage, one convolution kernel is applied independently to each input channel. Each channel is therefore filtered only within its own spatial domain. In the point-wise stage, the resulting feature maps are combined across channels using a 1×1 convolution, which integrates channel-wise information.
  Given the output feature map from the previous AFF module, denoted as $X^{l}\in R^{H\times W\times C}$, the DS block first applies spatial filtering to each channel independently. This is done through depthwise convolution:
  (1) $$X_{c}^{d}=X_{c}^{l}*K_{c}^{d},\;c=1,2,\ldots ,C$$
  where $K_{c}^{d}\in R^{k\times k}$ is the kernel for the *c*-th channel and ∗ denotes convolution. The resulting feature map $X^{d}\in R^{H\times W\times C}$ is then processed by pointwise convolution to fuse channel-wise information:
  (2) $$X^{p}=X^{d}*K^{p},\;K^{p}\in R^{1\times 1\times C\times C}$$
  To clarify the motivation for using depthwise separable convolution in the AFF module, Table 1 compares it with standard convolution in terms of structure, computational cost, and functional advantages.
  As shown in Table 1, DS convolution separates spatial filtering from channel mixing, which substantially reduces both parameter count and computational cost. This design makes the AFF module more lightweight while preserving essential feature representations. By learning spatial and channel-wise information separately, DS convolution also offers a more flexible feature-extraction mechanism.
  It is also worth discussing the possibility that standard convolution may yield less accurate results than DS convolution in certain scenarios, despite having a larger parameter space. In medical image analysis, training datasets are often limited in size compared with natural image benchmarks. Standard convolution jointly learns spatial and channel-wise features with K×K×M×N parameters, which substantially increases the risk of overfitting when training data is scarce [36]. In contrast, DS convolution factorizes this operation into separate spatial and channel-wise stages, effectively acting as a structural regularizer that constrains the hypothesis space. This factorization has been shown to improve generalization on small-scale datasets [37]. Furthermore, prior studies on lightweight architectures have demonstrated that models employing DS convolution can match or even surpass the accuracy of their standard convolution counterparts when the training set is limited [38]. In the context of breast ultrasound analysis, where annotated data is inherently scarce, this regularization effect is particularly beneficial, enabling the AFF module to learn more robust and transferable feature representations without overfitting to training-specific noise.
2. SE module: The AFF module incorporates the SE (Squeeze-and-Excitation) module [39], which adaptively modifies the inter-channel weights by comprehending the dependencies of global features. The SE module consists of a squeezing operation, generating a comprehensive description of the feature channel via global average pooling, and an excitation operation, learning the non-linear relationship through fully connected layers and emitting the weights for each channel. These weights function to intensify or suppress specific channel features, thereby elevating the accuracy of feature fusion.
  The output $X^{p}$ is subsequently refined by an SE block to emphasize informative channels and suppress less relevant ones. First, global average pooling is applied to aggregate context:
  (3) $$z_{c}=\frac{1}{H\times W}\sum_{i=1}^{H}\sum_{j=1}^{W}X_{c}^{p}\left(i,j\right)$$
  Then, two fully connected layers with ReLU and sigmoid activations generate channel-wise attention weights:
  (4) $$s=\sigma (W_{2}\cdot \delta \left(W_{1}\cdot z\right))$$
  The recalibrated feature map is obtained by reweighting each channel:
  (5) $$X_{c}^{SE}=s_{c}\cdot X_{c}^{p}$$
3. Feature concatenation: The feature map acquired via the depth-wise separable convolution is merged with the corresponding feature map on the primary pathway. This process amalgamates features from varying levels, increasing the depth of the model’s feature representation.
  The main-branch feature from the downsampling path is denoted as $X^{U}\in R^{H\times W\times C}$. This is concatenated with the recalibrated side-branch feature $X^{SE}$:
  (6) $$X^{cat}=\mathrm{Concat}\left(X^{SE},X^{U}\right)$$
  To fuse the concatenated features, a convolutional layer followed by batch normalization and ReLU is applied:
  (7) $$X^{fused}=\mathrm{ReLU}\left(\mathrm{BN}\left(\mathrm{Conv}\left(X^{cat}\right)\right)\right)$$
  An additional SE block is applied to $X^{fused}$ to further enhance channel-wise discriminability, and the final output is processed by a nonlinear mapping:
  (8) $$X^{AFF}=\mathrm{ReLU}\left(\mathrm{BN}\left(\mathrm{Conv}\left(X_{fused}^{SE}\right)\right)\right)$$
  In the actual implementation, the AFF side branch uses a 3×3 depthwise convolution followed by a 1×1 pointwise convolution. The fusion operations in Equations (7) and (8) use 3×3 kernels. In addition, the AFF branch applies dropout with a rate of 0.2, and the embedded SE units use two fully connected layers with a reduction ratio of 16. Equivalently, for an AFF feature with channel dimension *C*, the two fully connected layers in each SE unit map the channel descriptor from *C* to C/16 and then back from C/16 to *C*.

Executing these steps, the AFF module can effectively escalate the representational efficacy of features and optimize the feature fusion procedure through adaptive approaches, offering robust feature support for downstream tasks like classification and segmentation.

### 3.3. Cross-Level Attention Enhancement Module (CLAE Module)

**Figure 3:**
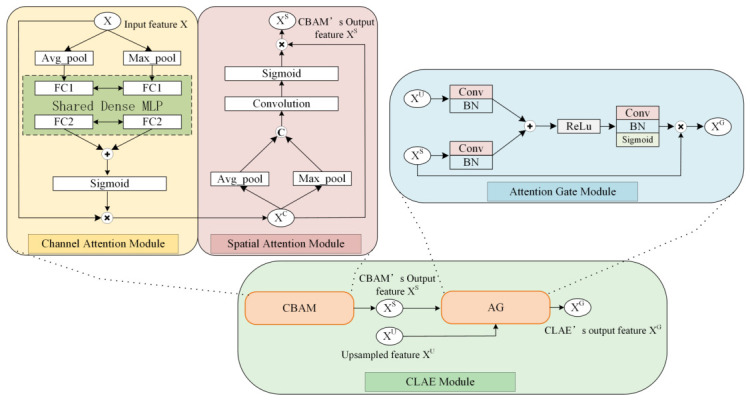
Cross-Level Attention Enhancement Module (CLAE Module). In this schematic, the yellow block denotes the channel attention module, the pink block denotes the spatial attention module, the blue block denotes the attention gate module, and the green block denotes the overall CLAE module. The arrows indicate the feature-flow direction, the dotted lines connect the enlarged submodule schematics to their positions in the overall CLAE module, and ⊗ denotes element-wise gating or feature reweighting.

A key feature of the N-Unet model is its CLAE module, which is specifically designed to enhance the quality and relevance of feature maps in the decoder. As shown in Figure 3, the CLAE module plays a crucial role in the network architecture.

Let the input feature map of the CLAE module be $X\in R^{H\times W\times C}$, and let $X^{U}$ denote the upsampled feature from the decoder. The first attention component is the convolutional block attention module (CBAM), which sequentially applies channel attention and spatial attention to adaptively refine features. Below is a detailed explanation of its three core components:

1. CBAM (Convolutional Block Attention Module): The first component of the CLAE module is CBAM, which guides attention in both the channel and spatial domains of the feature map. In the channel attention process, CBAM captures global context information by using global average pooling and global max pooling. It then generates channel attention maps through two independent fully connected layers. This ensures that the model can emphasize the feature channels most important for the current task. In the spatial attention process, CBAM further highlights important areas in the image based on the output of channel attention. It uses a small convolutional kernel to process the attention map, thus emphasizing the model’s focus on key positions.
  For the channel attention step, average pooling and max pooling are applied over spatial dimensions to generate descriptors:
  (9) $$z^{avg}=\frac{1}{H\times W}\sum_{i,j}X\left(i,j\right),\;z^{max}=\max_{i,j}X\left(i,j\right)$$
  These are passed through a shared multilayer perceptron (MLP), composed of two fully connected layers, and summed:
  (10) $$s^{c}=\sigma \left(\mathrm{MLP}\left(z^{avg}\right)+\mathrm{MLP}\left(z^{max}\right)\right)$$
  The channel-refined feature $X^{C}$ is computed as:
  (11) $$X^{C}=s^{c}\cdot X$$
  For spatial attention, CBAM uses average pooling and max pooling along the channel axis, followed by a convolutional layer:
  (12) $$M^{S}=\sigma \left(f^{7\times 7}\left(\left[\mathrm{AvgPool}\left(X^{C}\right);\mathrm{MaxPool}\left(X^{C}\right)\right]\right)\right)$$
  The final CBAM output is obtained by:
  (13) $$X^{S}=M^{S}\cdot X^{C}$$
2. AG (Attention Gate): As the second component of the CLAE module, AG gates the feature maps on the upsampling path. AG uses attention coefficients, calculated from the feature map of the previous layer and the feature map in the skip connection, to dynamically adjust the weights of each feature. This mechanism allows the model to suppress irrelevant areas while emphasizing target areas. Let $X^{S}$ be the output of CBAM and $X^{U}$ the upsampled feature. These are processed by convolutional layers and summed:
  (14) $$\alpha =\sigma (W_{\alpha }\cdot \delta \left(W_{1}X^{S}+W_{2}X^{U}\right))$$
  where $W_{1},W_{2}\in R^{1\times 1\times C\times F}$ and $W_{\alpha }\in R^{1\times 1\times F\times 1}$ are learnable convolutional weights, with *F* denoting the intermediate channel number. δ(·) and σ(·) denote ReLU and Sigmoid activations, respectively. The gated feature is obtained by:
  (15) $$X^{G}=\alpha \cdot X^{S}$$
  In implementation, CBAM channel attention is generated by a shared two-layer MLP with a reduction ratio of 16, which maps the channel descriptor from *C* to C/16, applies a ReLU activation, and then projects it back to *C*. The same MLP is shared by the average-pooled and max-pooled channel descriptors. Its spatial attention branch uses a 7×7 convolution. In the attention gate, the projections $W_{1}$, $W_{2}$, and $W_{\alpha }$ are implemented with 1×1 convolutions, matching the practical decoder-side realization of Equation (14).
  As a decoder-side refinement block, the CLAE module bridges high-level semantic and low-level spatial features. Its dual-attention design and feature fusion pathway enhance detail preservation and foreground focus, especially in complex medical image regions.

Throughout the entire N-Unet architecture, the CLAE module acts as a bridge, improving both the quality of the feature map and the preservation of details and context information during the upsampling process. This design allows the N-Unet to maintain a high level of sensitivity when processing complex medical images.

### 3.4. Conditional Segmentation Boosting (CSB Module)

**Figure 4:**
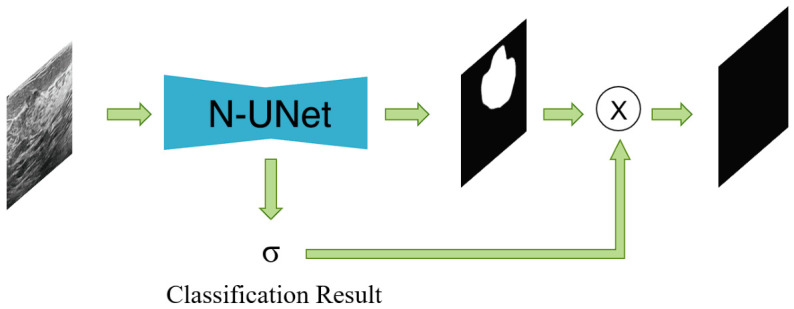
Conditional Segmentation Boosting (CSB Module). Here, σ denotes the discrete binary classification decision, the green arrows indicate the forward information flow from the input image through N-Unet and the routing of the classification result to the gating node, and the multiplication symbol denotes scalar gating of the segmentation map by (1−σ); thus, σ=0 preserves the segmentation output, whereas σ=1 suppresses it.

The CSB module is a post-processing refinement block that incorporates the classification result into the segmentation output. As shown in Figure 4, it uses the classification branch of N-Unet to selectively suppress predicted segmentation regions on images classified as normal.

The classification branch predicts whether the input breast ultrasound image contains a nodule. The output σ∈{0,1} is a discrete binary value, where σ=0 indicates a nodule-containing image and σ=1 indicates a normal image. Practically, the classification branch first applies global average pooling to the bottleneck feature and then uses a two-layer fully connected head with dimensions 512→1024→2. The resulting binary decision is used as the gating signal in the CSB module.

Let $S\in R^{H\times W}$ be the raw segmentation prediction. The CSB module computes the final segmentation output $\hat{S}$ as:

(16) $$\hat{S}=\left(1-\sigma \right)\cdot S$$

Here, (1−σ) acts as a binary scalar gate on the whole segmentation map rather than a probability weight. Therefore, when σ=0, the segmentation output is preserved ($\hat{S}=S$), whereas when σ=1, the segmentation output is fully suppressed ($\hat{S}=0$). In Figure 4, the multiplication symbol denotes this scalar gating operation rather than a tensor product between two dense outputs.

Under this formulation, the segmentation output is preserved only when the classification result indicates the presence of a nodule. For images classified as negative, the segmentation map is fully suppressed, which removes semantically inconsistent foreground predictions. At the same time, this hard binary gate implies that a false-negative classification will also suppress a true lesion segmentation. We therefore treat this as an explicit limitation of the current CSB design and analyze it further in Section 5.4.

This binary gating strategy links the two tasks through a simple decision-consistency rule. The CSB module introduces no additional parameters and improves the semantic consistency of the final prediction. Accordingly, CSB should be understood as a classification-conditioned consistency module rather than a standalone segmentation enhancer: its practical effectiveness depends on the reliability of the classification branch, while the information flow remains strictly one-way from classification to segmentation.

### 3.5. Adaptive Multi-Task Learning Loss (AMTL Loss)

Within N-Unet, we introduce the AMTL loss to optimize classification and segmentation jointly. Rather than relying on manually tuned task weights or a direct sum of task losses, AMTL uses task uncertainty to balance the contributions of the two branches automatically. This mechanism allows the model to adapt its emphasis during training, which is particularly useful in medical image analysis, where task difficulty can vary substantially across samples.

The AMTL loss consists of two components: the classification loss ($L_{\mathrm{FocalBCE}}$) and the segmentation loss ($L_{\mathrm{seg}}$). Their detailed formulations are given below.

#### 3.5.1. Classification Loss Function

Binary cross-entropy (BCE) loss is commonly used in binary classification tasks. It measures the difference between predicted probabilities and ground-truth labels. The standard BCE loss is defined as:

(17) $$L_{\mathrm{BCE}}=-\sum_{i=1}^{N}\left[\xi_{i}\log\left(\zeta_{i}\right)+\left(1-\xi_{i}\right)\log\left(1-\zeta_{i}\right)\right]$$

Here, $\zeta_{i}\in \left(0,1\right)$ is the predicted probability for the *i*-th sample, $\xi_{i}\in \left\{0,1\right\}$ is the ground-truth label, and *N* is the number of samples.

To address class imbalance, a weighted BCE loss is often adopted. It assigns higher loss weights to underrepresented classes. The weighted version is expressed as:

(18) $$L_{\mathrm{wBCE}}=-\sum_{i=1}^{N}\left[\omega_{i}\xi_{i}\log\left(\zeta_{i}\right)+\left(1-\xi_{i}\right)\log\left(1-\zeta_{i}\right)\right]$$

where $\omega_{i}=\frac{N^{\mathrm{neg}}}{N^{\mathrm{pos}}}$ is the lesion-class weight computed for the binary lesion-presence task. Before computing $\omega_{i}$, benign and malignant samples are merged into a single lesion-containing class, whereas normal samples form the other class. Accordingly, $N^{\mathrm{pos}}$ and $N^{\mathrm{neg}}$ denote the numbers of lesion-containing and normal samples, respectively, in the training subset of each fold.

While weighted BCE addresses class imbalance, it does not differentiate between easy and hard samples. To further mitigate this, Focal Loss [35] was introduced to reduce the loss contribution from well-classified samples, allowing the model to focus more on hard misclassified instances. The general form of focal loss for binary classification is:

(19) $$L_{\mathrm{FL}}=-\sum_{i=1}^{N}\alpha_{i}{\left(1-p_{t}^{\left(i\right)}\right)}^{\gamma }\log\left(p_{t}^{\left(i\right)}\right)$$

where

$$p_{t}^{\left(i\right)}=\left\{\begin{array}{cc} \zeta_{i} & \mathrm{if}\;\xi_{i}=1\\ 1-\zeta_{i} & \mathrm{if}\;\xi_{i}=0 \end{array}\right.$$

and $\alpha_{i}$ is a weighting factor for class balancing (optional), and γ≥0 is the focusing parameter. A higher γ reduces the loss assigned to easy samples.

In our work, we combine the ideas of weighted BCE and focal loss into a unified Focal-BCE loss. Specifically, we introduce the weight $\omega_{i}$ into the focal loss formulation to enhance the model’s focus on both hard and minority class samples:

(20) $$L_{\mathrm{FocalBCE}}=-\sum_{i=1}^{N}\omega_{i}{\left(1-p_{t}^{\left(i\right)}\right)}^{\gamma }\log\left(p_{t}^{\left(i\right)}\right)$$

This formulation addresses both class imbalance (through $\omega_{i}$) and sample difficulty (through γ), thereby improving classification robustness in highly imbalanced settings such as breast ultrasound diagnosis. In our experiments, the focusing parameter γ is fixed at 2, following the standard focal-loss setting. This choice provides a moderate emphasis on hard misclassified samples without excessively amplifying unstable samples in relatively small BUS datasets. Meanwhile, $\omega_{i}$ is not manually tuned but computed separately in each training fold from $N^{\mathrm{neg}}/N^{\mathrm{pos}}$ in the binary lesion-presence label space, so that the classification loss remains adaptive to fold-specific class imbalance.

#### 3.5.2. Segmentation Loss Function

For the segmentation task, we adopt Dice loss, which measures the similarity between the predicted mask and the ground-truth mask. This loss is well suited to medical image segmentation because it directly emphasizes spatial overlap. The formulation is as follows:

(21) $$L_{\mathrm{seg}}=1-\frac{2\times \sum_{i=1}^{N}p_{i}\times g_{i}+\epsilon }{\sum_{i=1}^{N}p_{i}+\sum_{i=1}^{N}g_{i}+\epsilon }$$

where $p_{i}$ represents the model’s prediction value for each pixel, $g_{i}$ is the corresponding actual label value, and ϵ is a small constant used to prevent the denominator from being zero. In this paper, it is set to $1\times 10^{-5}$.

#### 3.5.3. Overall Loss Function

Traditionally, multi-task loss functions combine task-specific losses using fixed weights:

(22) $$L\left(\theta \right)=\sum_{d=1}^{D_{s}}\lambda_{d}L_{d}\left(\theta \right)$$

where $\lambda_{d}$ denotes the manually chosen weight for each task. However, these hyperparameters are often difficult to tune and do not adapt to changing task uncertainties during training.

Inspired by Kendall et al. [12], we adopt a homoscedastic uncertainty-based weighting strategy. The overall AMTL loss is defined as:

(23) $$\begin{array}{cc} L_{\mathrm{AMTL}}\left(\theta \right)= & \frac{1}{2\sigma_{1}^{2}}L_{\mathrm{FocalBCE}}\left(\theta \right)+\frac{1}{2\sigma_{2}^{2}}L_{\mathrm{seg}}\left(\theta \right)\\ \; & +\log\left(\sigma_{1}\right)+\log\left(\sigma_{2}\right) \end{array}$$

Here, $\sigma_{1}$ and $\sigma_{2}$ are learnable uncertainty variables for classification and segmentation tasks, respectively. These values are optimized jointly with the network parameters, allowing for automatic task-wise loss reweighting throughout training. For reproducibility, the uncertainty parameters are initialized as $\sigma_{1}=1.0$ and $\sigma_{2}=1.0$ before training, and are then optimized jointly with the network parameters throughout the full training process.

In other words, the task weights are already included in the optimization process implicitly. The effective weighting coefficients in Equation (23) are $\frac{1}{2\sigma_{i}^{2}}$, and since $\sigma_{i}$ is learned jointly with the network parameters, the model automatically updates the corresponding task weights during backpropagation. Compared with directly optimizing unconstrained coefficients $\lambda_{d}$ in Equation (22), this uncertainty-based parameterization is more stable because the $\log(\sigma_{i})$ term regularizes the learned weights and helps avoid degenerate solutions in which one task is trivially suppressed [12].

In multi-task learning, especially when more tasks are involved, the total loss can become biased toward tasks with larger loss magnitudes or gradient scales. Although uncertainty-based weighting helps alleviate this issue, it does not completely remove imbalances caused by differences in task density or loss range.

To further mitigate this issue, we normalize the aggregated loss by the number of tasks $D_{s}$, so that the final value reflects the average contribution across tasks. This normalization prevents tasks with large losses or high uncertainty from dominating optimization and promotes more stable convergence. The final loss is given as:

(24) $$L_{\mathrm{AMTL}}=\frac{1}{D_{s}}\sum_{i=1}^{D_{s}}\left(\frac{1}{2\sigma_{i}^{2}}L_{i}+\log\sigma_{i}\right)$$

This formulation encourages each task to contribute more evenly in expectation and reduces the risk that one task will dominate the optimization process.

#### 3.5.4. Summary of the Loss Function

To optimize classification and segmentation jointly, we design the AMTL loss by combining task-specific objectives with a dynamic uncertainty-based weighting mechanism. Specifically, we use Focal-BCE for classification to address class imbalance and sample difficulty, and Dice loss for segmentation to emphasize spatial overlap. These losses are combined through homoscedastic uncertainty weighting, allowing the network to learn task-specific confidence values and balance their influence automatically during training. We further normalize the total loss by the number of tasks to promote stable optimization across objectives.

This loss formulation adapts to variations in task difficulty and data imbalance while eliminating the need for manual task-weight tuning. As a result, it provides a more robust and efficient training process for complex multi-task settings such as breast ultrasound analysis, where both classification and fine-grained segmentation are important.

## 4. Results

In this section, we present the experimental setup and results. First, we introduce the dataset and the experimental platform. Second, we present the parameter settings and evaluation metrics. Finally, we present the comparative experiments and ablation results.

### 4.1. Dataset

**Figure 5:**
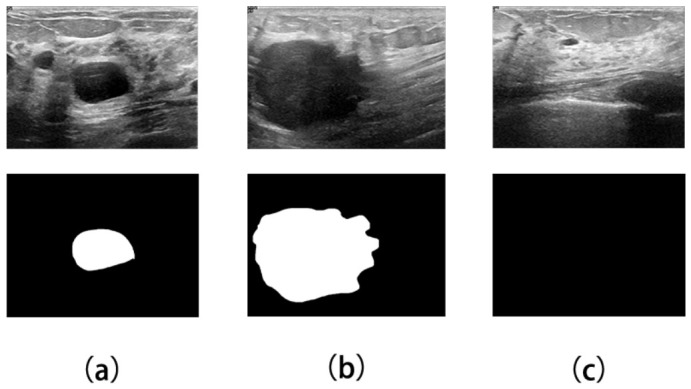
Examples of breast ultrasound images and their corresponding segmentation masks from the Breast Ultrasound Image Dataset (BUSI). (**a**) benign lesions and their corresponding mask images, (**b**) malignant lesions and their corresponding mask images, and (**c**) normal breast tissue without lesions and their corresponding mask images.

The first dataset used in this study is the Breast Ultrasound Image (BUSI) dataset, which was collected in 2018 and reported by Al-Dhabyani et al. [40], as shown in Figure 5. The dataset contains 780 ultrasound images from female patients aged 25 to 75 years. The images are provided in RGB PNG format with an average size of 500×500 pixels, together with corresponding grayscale ground-truth (GT) masks. The dataset is divided into three categories: benign, malignant, and normal, with 437, 210, and 133 images, respectively. In the GT masks, lesion regions are shown in white against a black background.

**Figure 6:**
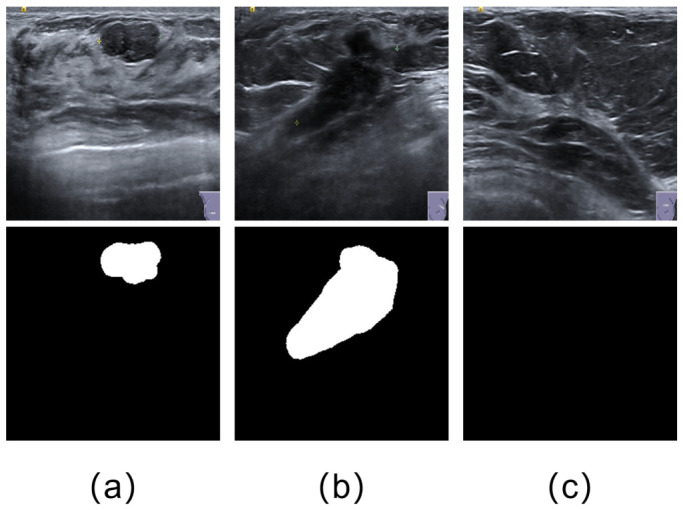
Sample images from the BUS-UCLM dataset. The first row presents the original ultrasound images, while the second row shows the binary ground-truth masks used in this study, where white denotes the lesion foreground and black denotes the background. (**a**) benign case and its corresponding binary mask, (**b**) malignant case and its corresponding binary mask, and (**c**) normal breast tissue without lesions and its corresponding binary mask.

As shown in Figure 6, the BUS-UCLM dataset contains 683 breast ultrasound images from 38 patients, collected between 2022 and 2023 using a Siemens ACUSON S2000^TM^ ultrasound system manufactured by Siemens Medical Solutions USA, Inc. (Mountain View, CA, USA). It includes 174 benign, 90 malignant, and 419 normal cases. The ground-truth masks are provided in RGB format, where green denotes benign lesions, red denotes malignant lesions, and black denotes background or normal breast tissue. For segmentation training and evaluation, the RGB annotations were converted into binary lesion masks by merging both green benign regions and red malignant regions into a single foreground class, while black pixels were treated as background. All subsequent mask resizing operations were performed with nearest-neighbor interpolation to preserve label discreteness and avoid boundary mixing. As the second dataset used in this study, BUS-UCLM introduces additional variation in imaging conditions and lesion characteristics, which helps assess cross-dataset robustness within the present breast ultrasound setting.

To maintain a consistent distribution of nodule sizes and categories across dataset subsets, all images and corresponding lesion masks were standardized to a resolution of 256×256 pixels. The lesion area *A* is defined as the number of foreground pixels in the resized mask. The nodules were then stratified into three groups according to *A*, defined as follows:

(25) $$\left\{\begin{array}{cc} A<\alpha , & \mathrm{small}\;\mathrm{nodules}\\ \alpha \leq A<\beta , & \mathrm{medium}\;\mathrm{nodules}\\ A\geq \beta , & \mathrm{large}\;\mathrm{nodules} \end{array}\right.$$

The stratification thresholds α and β are determined from the area distribution of lesion-positive samples only (i.e., samples with A>0), where α and β correspond to the empirical 33% and 66% quantiles, respectively. For the BUSI dataset, α=2097 pixels and β=6725 pixels; for the BUS-UCLM dataset, α=1811 pixels and β=4672 pixels. This design avoids distortion from zero-area normal samples and yields a more balanced three-tier lesion size partition.

Based on this stratification, we adopted a five-fold cross-validation protocol. In each fold, the data was divided into training, validation, and test sets using an approximate ratio of 6:2:2. This strategy preserves the distributions of lesion size and category across the three subsets and helps reduce bias caused by sample imbalance. Here, the size stratification is used to stabilize fold construction and preserve comparable lesion-size distributions across subsets; it is not itself a substitute for dedicated size-wise performance analysis.

### 4.2. Image Preprocessing

It should be noted that dataset splitting strictly precedes any data augmentation. The full dataset is first partitioned into training, validation, and test subsets at the image level. Augmentation is subsequently applied exclusively to the training subset, whereas the validation and test subsets undergo only deterministic preprocessing (resizing and normalization) with no random transformations. In each fold, no augmented derivative of any validation or test image is ever introduced into the training process. This design ensures complete isolation of the validation and test sets and eliminates any risk of data leakage between training and evaluation partitions.

We applied a sequence of preprocessing steps to the breast ultrasound images. For the training subset only, random resizing with bicubic interpolation was first used to scale images while preserving aspect ratio. For the GT masks, nearest-neighbor interpolation was used to preserve clear segmentation boundaries. After resizing, random rotations of $90^{\circ }$, $180^{\circ }$, or $270^{\circ }$ were applied to simulate different imaging orientations. Additional small random rotations within $\left[-10^{\circ },10^{\circ }\right]$ were used to increase data diversity further. Center cropping was then applied to obtain a more focused view of the region of interest. Horizontal and vertical flipping were also optionally used to simulate different imaging conditions. Together, these augmentations help the model learn more robust features and reduce sensitivity to image orientation. However, these operations provide only generic spatial diversity during training and do not explicitly simulate ultrasound-specific intensity variation, speckle noise, or other acquisition-dependent artifacts.

Finally, all images and GT masks were resized to the required network input size and converted into tensor format, completing the preprocessing pipeline.

### 4.3. Experimental Platform

All deep learning experiments were conducted in PyTorch 1.12.0 on a GPU server running Ubuntu 18.04. The server was equipped with an Intel i7-8700K CPU, 64 GB of RAM, and an Nvidia RTX 1080Ti GPU with 11 GB of memory. PyTorch was selected for its flexibility and efficient GPU support, which enabled stable training and evaluation of the proposed models.

Overall, this experimental platform provided an efficient environment for training and evaluating the deep learning models used in this study.

### 4.4. Parameter Settings

The main training settings were as follows: batch size =6, number of epochs =800, optimizer = Adam, initial learning rate $=1\times 10^{-5}$, and learning-rate schedule = cosine annealing.

Adam was selected because it is one of the standard optimizers for deep neural network training and is well suited to stochastic mini-batch optimization with heterogeneous gradient scales across tasks [41]. Although L-BFGS-B is a powerful quasi-Newton method for full-batch or near-deterministic optimization problems [42], it is not necessarily more effective for the present deep multi-task setting. Under mini-batch training, its curvature approximation is typically less reliable, and its memory and computational overhead are higher than those of Adam. Therefore, Adam provides a more practical and stable choice for optimizing the proposed N-Unet model.

### 4.5. Evaluation Metrics

#### 4.5.1. Evaluation Metrics for Classification

In this study, classification is formulated as a binary lesion-presence task. The positive class (y=0) denotes images containing nodules (benign or malignant), and the negative class (y=1) denotes normal breast images without nodules. Under this setting, precision and recall describe lesion detection rather than normal-case identification. We report accuracy, precision, recall, and F1-score as follows:

(26) $$\mathrm{acc}=\frac{tp+tn}{tp+fp+tn+fn}$$

(27) $$\mathrm{pre}=\frac{tp}{tp+fp}$$

(28) $$\mathrm{rec}=\frac{tp}{tp+fn}$$

(29) $$f1=2\times \frac{\mathrm{pre}\times \mathrm{rec}}{\mathrm{pre}+\mathrm{rec}}$$

where tp, fp, tn, and fn denote true positives, false positives, true negatives, and false negatives, respectively. Because the positive class corresponds to lesion-containing images, higher recall indicates fewer missed nodules, whereas higher precision indicates fewer normal images incorrectly classified as lesion-containing.

#### 4.5.2. Evaluation Metrics for Segmentation

We evaluate segmentation using Intersection over Union (IOU), Dice coefficient (DC), pixel accuracy (Acc), sensitivity (SE), and pixel precision (PC). All images are processed by the segmentation branch and refined by the classification-guided CSB module. The reported segmentation metrics are computed on a per-image basis and then averaged. In this study, normal cases with empty masks are excluded from all reported segmentation metrics. This is because empty-mask normal images contain no foreground pixels, so IOU, Dice, SE, and PC become undefined or convention-dependent when the prediction is also empty, whereas Acc can become background-dominated. Accordingly, the reported segmentation scores should be interpreted as lesion-delineation performance on lesion-containing images rather than as whole-cohort screening metrics. Here, TP, FP, TN, and FN denote pixel-level true positives, false positives, true negatives, and false negatives, respectively. The corresponding formulas are given in Equations (30)–(34).

(30) $$\mathrm{IOU}=\frac{TP}{TP+FP+FN}$$

(31) $$\mathrm{DC}=\frac{2TP}{2TP+FP+FN}$$

(32) $$\mathrm{Acc}=\frac{TP+TN}{TP+FP+TN+FN}$$

(33) $$\mathrm{SE}=\frac{TP}{TP+FN}$$

(34) $$\mathrm{PC}=\frac{TP}{TP+FP}$$

Together, these metrics provide a comprehensive evaluation of segmentation quality from both overlap-based and pixel-level perspectives.

### 4.6. Comparative Experiments

To evaluate the proposed N-Unet comprehensively, we compared it with several classic and state-of-the-art deep learning models for classification and segmentation. The comparative set includes Unet, ResUnet, M-Unet, AGUnet, ANUnet, several recent architectures, and the Transformer-based models UNETR and SwinUnet. All experiments were conducted on the BUSI and BUS-UCLM datasets under consistent dataset, hardware, and software settings to ensure fair comparison.

All main quantitative comparison tables in this section report results as mean ± standard deviation across evaluation folds to reflect performance stability. We adopt this unified presentation to provide a concise view of variability across models and datasets. While confidence intervals and formal statistical tests could offer further statistical characterization, the comparative discussion in the present manuscript is stated conservatively, and the observed performance differences are interpreted as empirical comparative results under the current evaluation setting.

**Figure 7:**
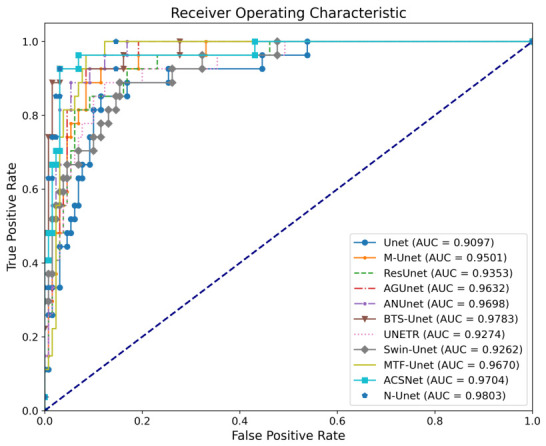
Classification task results on the BUSI dataset: the receiver operating characteristic (ROC) curve, where the purple dashed diagonal line denotes the chance-level reference (random-classifier) baseline.

**Figure 8:**
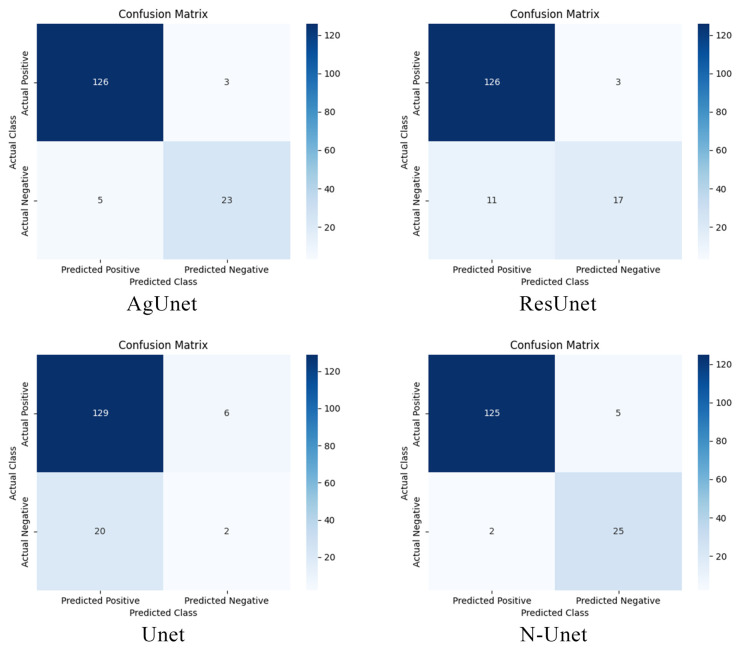
Confusion matrices for AGUnet, ResUnet, Unet, and N-Unet on the BUSI dataset.

For classification, the assessment focused on precision, recall, F1-score, and accuracy. Because accuracy alone can be misleading under class imbalance, classification performance was interpreted jointly with ROC-AUC and confusion-matrix behavior. The classification task is formulated as a binary lesion-presence problem (lesion-containing vs. normal), with positive/negative ratios of approximately 4.86:1 on BUSI and 1.59:1 on BUS-UCLM. Accordingly, Table 2 and Table 3 jointly report precision, recall, F1-score, and accuracy; Figure 7 reports ROC curves and AUC; and Figure 8 presents confusion matrices to show false-positive and false-negative behavior directly. In addition, Focal-BCE with the class-frequency weight $\omega_{i}$, as described in Section 3.5.1, is used during training to mitigate the effect of class imbalance. For segmentation, sensitivity, pixel accuracy, DC, and IOU were used to measure the models’ effectiveness in delineating breast nodule boundaries. These metrics provided a comprehensive evaluation of model performance and insights into their ability to capture fine details of lesion areas.

#### 4.6.1. Performance Analysis for Classification Task

To evaluate the classification performance of N-Unet comprehensively, we conducted comparative experiments on both the BUSI and BUS-UCLM datasets. The detailed results are summarized in Table 2 and Table 3, covering precision, recall, F1-score, and accuracy.

As shown in Table 2, N-Unet achieves the highest precision at 98.43%, with ANUnet ranking second at 97.64%. UNETR attains the highest recall at 99.22%; however, its lower precision and accuracy indicate a stronger tendency to classify normal samples as lesion-containing, resulting in more false positives. By contrast, N-Unet maintains a better-balanced classification profile, achieving an F1-score of 97.28% and the highest accuracy of 96.54%. As in the evaluation protocol above, this classification result is interpreted jointly with ROC-AUC and confusion-matrix behavior rather than by accuracy alone.

To further assess discrimination ability, we analyzed the ROC curves for all models, as shown in Figure 7. The area under the curve (AUC) quantifies each model’s ability to distinguish nodule-containing images from normal images across different thresholds. N-Unet achieves the highest AUC of 0.9803, confirming its strong discriminative power. Other high-performing models include BTS-Unet (0.9783), ANUnet (0.9698), and AGUnet (0.9632), whereas the traditional Unet records the lowest AUC at 0.9097.

To better understand classification tendencies, we examined the confusion matrices in Figure 8. N-Unet achieves 125 true positives while maintaining the highest true negative count of 25, indicating stronger control of false-positive errors. Although the traditional Unet correctly identifies 129 nodule-containing samples, it also produces substantially more false positives. In comparison, N-Unet offers a more reliable balance between sensitivity and specificity.

To further assess cross-dataset robustness within the present BUS setting, we evaluated classification performance on the BUS-UCLM dataset, with results summarized in Table 3. Compared with BUSI, N-Unet performs slightly worse on BUS-UCLM, particularly in recall (90.62%) and F1-score (93.55%). Transformer-based models such as UNETR (98.99% recall, 94.69% F1-score) and SwinUnet (98.00% recall, 94.23% F1-score) outperform N-Unet on these two metrics, indicating stronger sensitivity to nodule-containing cases.

Even so, N-Unet remains the most balanced model among the evaluated architectures. Although UNETR and SwinUnet achieve the highest recall, their lower precision (90.74%) indicates a stronger tendency to misclassify normal samples as lesion-containing. This trade-off reduces missed detections but increases the false-positive rate. By contrast, N-Unet maintains a better balance between sensitivity and specificity while retaining competitive recall.

The exceptional recall performance of UNETR can be attributed to its strong global feature aggregation capability. As a transformer-based architecture, UNETR leverages self-attention mechanisms to model long-range dependencies, enabling it to capture subtle differences between normal and abnormal cases. This allows it to detect nearly all nodules, but at the cost of reduced precision, as its decision boundary may become overly inclusive. Additionally, UNETR’s encoder-decoder structure with global attention might provide an advantage in small-scale datasets like BUS-UCLM, where local spatial relationships alone may be insufficient for robust classification.

Overall, the combined experimental results from the BUSI and BUS-UCLM datasets indicate that N-Unet remains robust across two breast ultrasound datasets with different data distributions. Across both datasets, N-Unet consistently ranks among the highest-performing models in terms of F1-score and accuracy. Unlike models such as UNETR and SwinUnet, which prioritize recall at the expense of precision, or ACSNet, which optimizes precision while sacrificing recall, N-Unet effectively maintains a stable balance between sensitivity and specificity. These findings support the potential clinical applicability of N-Unet within the present BUS setting. However, broader external generalization, including transfer to ultrasound datasets from other anatomical regions, remains to be validated.

#### 4.6.2. Performance Analysis for Segmentation Task

In the segmentation evaluation, as presented in Table 4 and Table 5, the N-Unet model demonstrates superior performance across multiple metrics. Particularly, it achieves the highest DC and IOU on both datasets, reflecting its strong capability in accurately segmenting breast nodules. The high Acc further validates its reliability in delineating nodule boundaries with minimal misclassification.

The BUSI segmentation results show that N-Unet outperforms the other architectures on the most important evaluation metrics. It achieves the highest Dice coefficient (0.8070) and IOU (0.7404), indicating a strong ability to capture lesion shape and boundary details. N-Unet also records the highest sensitivity (0.8114) and the highest pixel-wise precision (0.8400), showing that it detects lesion pixels effectively while limiting false positives. Although MTF-Unet achieves the highest pixel accuracy (0.9736), its Dice coefficient and IOU remain lower than those of N-Unet, suggesting weaker boundary delineation.

Among other models, ANUnet and ACSNet perform relatively well, with Dice coefficients of 0.7854 and 0.7846, respectively, indicating their effectiveness in capturing nodule regions. However, their lower IOU values suggest potential segmentation inconsistencies, particularly in cases with irregular boundaries. Traditional models such as Unet and ResUnet show lower performance across all metrics, highlighting their limitations in dealing with the complexity of breast nodules, particularly in distinguishing benign and malignant lesions. Overall, the results on the BUSI dataset demonstrate that N-Unet provides the most balanced and accurate segmentation performance, excelling in both sensitivity and pixel-wise precision while effectively delineating nodule boundaries.

The BUS-UCLM segmentation results, presented in Table 5, confirm the strong performance of N-Unet on a different dataset with distinct imaging characteristics. N-Unet achieves the highest Dice coefficient (0.9216) and IOU (0.8974), outperforming all competing models. It also attains the highest pixel-wise precision (0.9215) and sensitivity (0.9329), indicating robust lesion capture with few false positives and false negatives. ACSNet also performs strongly (DC = 0.9115, IOU = 0.8857), but its slightly lower sensitivity (0.9239) suggests mild under-segmentation in some cases, especially for malignant nodules with highly irregular boundaries.

By contrast, UNETR (DC = 0.7283, IOU = 0.6985) and SwinUnet (DC = 0.7564, IOU = 0.6879) perform notably worse on BUS-UCLM, indicating that, under the present experimental setting, these Transformer-based baselines were less effective than the better-performing CNN-based models in this study at preserving precise lesion boundaries. A plausible interpretation is that their heavier reliance on global-context modeling and greater sensitivity to limited training data may compromise fine-grained boundary recovery in this relatively data-constrained setting. In ultrasound segmentation, where lesion margins are often weak, noisy, and irregular, such behavior may lead to less accurate delineation.

Accordingly, this interpretation should be regarded as a cautious interpretation rather than a confirmed causal conclusion. The current study establishes the observed performance gap, but it does not determine whether that gap is mainly related to architectural inductive bias, data regime, or optimization strategy. Dedicated experiments that vary training-set size and Transformer-specific optimization strategies will be needed to assess this interpretation more directly.

**Figure 9:**
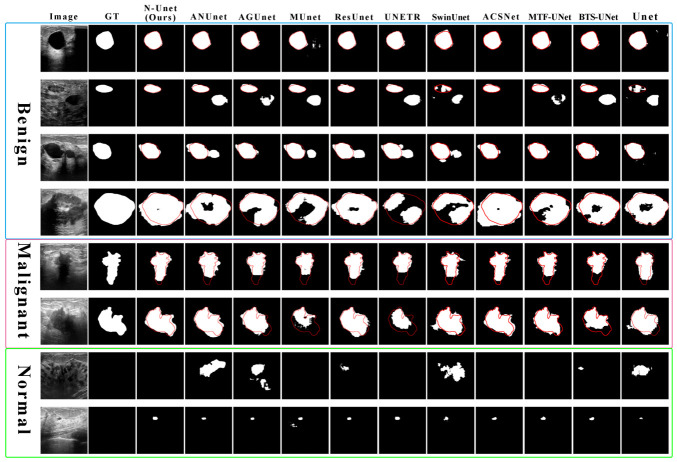
Qualitative comparison of breast nodule segmentation results on the BUSI dataset. The first column shows the original ultrasound images, and the second column (GT) shows the manually annotated ground-truth masks. The remaining columns present results from N-Unet, ANUnet, AGUnet, M-Unet, ResUnet, UNETR, SwinUnet, ACSNet, MTF-Unet, BTS-Unet, and Unet. The rows correspond to benign nodules, malignant nodules, and normal breast images. Red contours indicate the ground-truth boundaries for visual comparison.

We also compared the qualitative segmentation performance of different models in Figure 9. The figure includes benign nodules, malignant nodules, and normal images without nodules. Each column presents the output of one model or the corresponding reference information, and the red contour marks the manually annotated ground-truth boundary for direct visual comparison.

N-Unet shows strong agreement with the ground truth in benign nodule segmentation, although minor off-contour predictions are visible in some cases. For malignant nodules, it captures the irregular lesion boundaries reasonably well despite small local deviations. For normal images without nodules, N-Unet also maintains high segmentation accuracy with very few spurious regions.

Compared with N-Unet, some models, including AGUnet, UNETR, MTF-Unet, and Unet, are more prone to mis-segmentation in benign cases. For malignant nodules, most models struggle more with boundary detail, likely because malignant lesions often have more irregular and complex contours than benign ones. In normal images, most methods avoid severe mis-segmentation, but some models, such as SwinUnet, still make visible errors. In the last normal example, all models incorrectly identify normal tissue as a lesion region. These observations show that even advanced deep learning models still have difficulty distinguishing nodules from normal tissue when clear morphological cues are absent.

**Figure 10:**
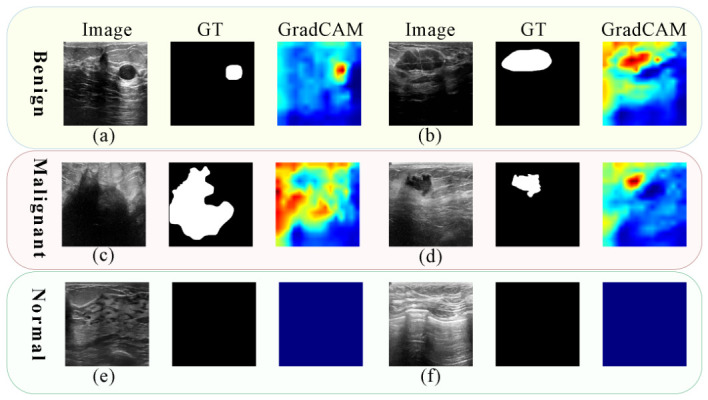
GradCAM visualization of N-Unet on breast ultrasound images. In each subfigure, the left panel shows the original ultrasound image, the middle panel shows the binary ground-truth (GT) mask, and the right panel shows the GradCAM heatmap generated by N-Unet. In the GT masks, white denotes lesion foreground and black denotes background. In the GradCAM heatmaps, warm colors (red/yellow) indicate higher model attention, whereas cool colors (green/blue) indicate lower attention. (**a**) first benign case; (**b**) second benign case; (**c**) first malignant case; (**d**) second malignant case; (**e**) first normal case without lesions; and (**f**) second normal case without lesions.

Figure 10 shows GradCAM heatmaps that visualize the image regions most associated with the N-Unet response during breast nodule recognition. These visualizations are intended as qualitative interpretability illustrations rather than quantitative evidence of precise lesion localization.

In the shown benign cases, the heatmap exhibits elevated activation around the lesion region, although the response is not strictly confined to the annotated boundary. For malignant nodules, the activation extends across both the lesion core and part of the surrounding tissue, consistent with the more irregular morphology and heterogeneous appearance of these cases. For normal images, the displayed examples show comparatively weaker and less concentrated activation.

We also observed that some N-Unet heatmaps contain highly activated regions outside the lesion itself. Such extra-lesion activation may reflect surrounding contextual cues, but it may also indicate imperfect localization or possible dependence on background tissue. Therefore, these GradCAM maps are not interpreted as inherently positive findings and should be treated only as coarse qualitative indications of model attention rather than as accurate delineations of lesion extent.

Overall, the GradCAM results provide an interpretable qualitative view of the regions associated with the model response. However, no dedicated quantitative evaluation of GradCAM localization or explanation faithfulness was conducted in the current study, so these visualizations are not used as quantitative validation of precise feature localization.

#### 4.6.3. Failure Case Analysis

**Figure 11:**
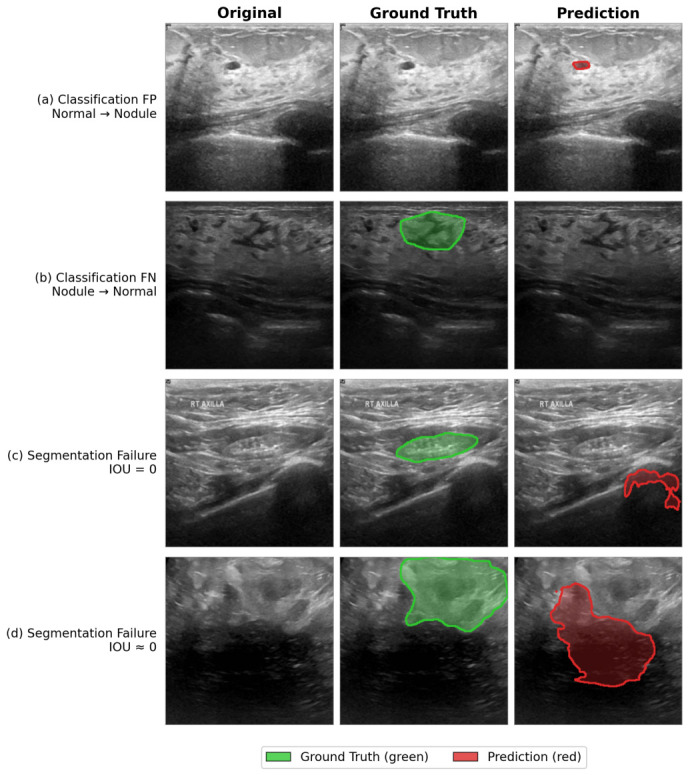
Representative failure cases of N-Unet on the BUSI dataset. Each row presents one failure scenario with three panels: original image (left), ground-truth annotation with green overlay (center), and model prediction with red overlay (right). (**a**) Classification false positive: a normal sample is misclassified as nodule-containing; the segmentation sub-network independently produces a spurious activation. (**b**) Classification false negative: a nodule-containing sample is misclassified as normal, causing CSB to suppress the entire segmentation output. (**c**) Segmentation failure with IOU = 0: the predicted region is completely displaced from the ground-truth lesion. (**d**) Segmentation failure with IOU ≈ 0: the predicted region is substantially misaligned relative to the ground-truth annotation.

To provide a more complete picture of model behavior, we further analyze representative failure cases, as illustrated in Figure 11. Four distinct failure modes are identified from the BUSI test set. Each case presents the original image, the ground-truth annotation (green overlay), and the model prediction (red overlay).

(a) Classification false positive (FP). A normal sample is incorrectly classified as nodule-containing. This type of error typically arises from ambiguous tissue textures that visually resemble nodule echogenicity. Under the CSB formulation ($\hat{S}=\left(1-\sigma \right)\cdot S$), a false-positive prediction (σ=0) preserves the segmentation output fully; whether any spurious activation follows depends on the segmentation sub-network’s own behavior, not on the CSB gate.
(b) Classification false negative (FN). A nodule-containing sample is incorrectly classified as normal. Visually, the annotated lesion region exhibits weak discriminability and shares substantial texture similarity with normal tissue patterns, without showing a prominent or typical nodule-like appearance. As a result, the classification branch fails to assign sufficient lesion confidence at the image level.
(c) Segmentation failure with IOU = 0. Classification is correct, but the predicted segmentation region is completely displaced from the ground-truth lesion. The predicted contour latches onto a high-contrast non-lesion structure, likely owing to ambiguous boundary cues in images containing multiple hyperechoic regions.
(d) Segmentation failure with IOU ≈ 0. Classification is correct, but the predicted region is substantially misaligned relative to the ground-truth annotation. The model captures a partial shape in the approximate vicinity of the lesion but fails to accurately delineate the true lesion boundary, likely attributable to the large size and irregular morphology of the target lesion.

These failure patterns collectively highlight three actionable limitations: (1) asymmetric propagation of classification errors into segmentation—false-negative predictions deterministically suppress the entire segmentation output via the CSB gate, while false-positive predictions conditionally yield spurious activations through independent sub-network behavior; (2) susceptibility to high-contrast non-lesion structures in multi-region images; and (3) reduced boundary delineation accuracy for large or morphologically irregular lesions. These observations motivate future work in improved classification robustness, adversarial feature disentanglement, and boundary-aware loss design.

### 4.7. Summary of Comparative Experiments

Across the classification experiments, N-Unet achieves the highest accuracy on both BUSI and BUS-UCLM while remaining among the strongest models in precision and F1-score. By contrast, Transformer-based models such as UNETR and SwinUnet attain higher recall on BUS-UCLM, but this gain is accompanied by lower precision and therefore more false-positive predictions. Overall, N-Unet provides the most balanced classification behavior among the compared methods.

For segmentation, N-Unet consistently achieves the best Dice coefficient and IOU on both BUSI and BUS-UCLM. Competitive CNN-based baselines such as ANUnet and ACSNet remain effective, but none match the consistency of N-Unet across the two datasets. In comparison, Transformer-based models are less effective at preserving fine lesion boundaries, suggesting that task-aware multi-scale fusion and decoder refinement remain important for breast ultrasound segmentation.

In addition to predictive performance, computational efficiency is important for practical deployment. We therefore compared the main competing methods in terms of parameter count and FLOPs. As summarized in Table 6, the reported complexity statistics were collected from confirmed model settings rather than recomputed with a single external profiling package. In our implementation, the N-Unet FLOPs value was computed with the THOP profiler for a single 1×3×256×256 input, consistent with the standardized network input resolution used in preprocessing. The reported FLOPs value should therefore be interpreted under this input size and profiling convention. Under this comparison, N-Unet requires 8.95 M parameters and 14.74 GFLOPs, giving it the most favorable efficiency profile among the evaluated methods. Because the compared architectures were not all originally proposed with identical joint-task design goals, this complexity comparison is intended as a practical efficiency reference for the evaluated model settings rather than as a standalone fairness claim about architectural design merit. The more direct evidence for the benefit of joint learning is provided separately by the dedicated N-Unet-Cls and N-Unet-Seg comparison reported later.

**Figure 12:**
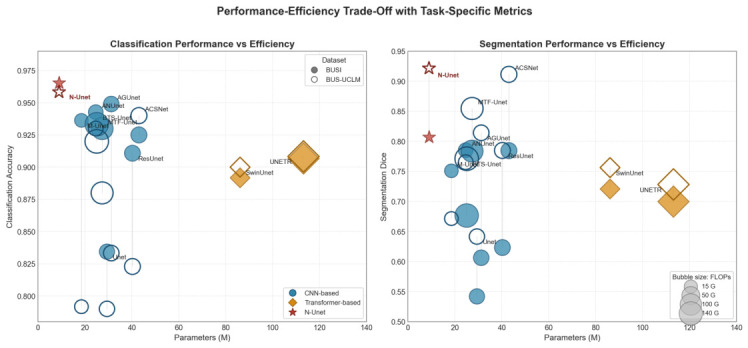
Task-specific performance-efficiency trade-off of different models. The horizontal axis denotes the number of parameters, the left panel reports classification accuracy, the right panel reports segmentation Dice, and the bubble size represents FLOPs. For each model, filled markers indicate BUSI results and hollow markers indicate BUS-UCLM results. Specifically for N-Unet, the filled five-point star denotes the BUSI result and the hollow five-point star denotes the BUS-UCLM result.

Figure 12 illustrates this trade-off more transparently using the original task-specific metrics rather than a post-hoc aggregated score. The revised visualization reports classification accuracy and segmentation Dice in separate panels while preserving the same efficiency dimensions, namely parameter count and FLOPs. This avoids imposing an equal-weighting assumption between classification and segmentation and enables direct task-level comparison across BUSI and BUS-UCLM. Under this view, N-Unet remains in the favorable low-complexity region while maintaining competitive or superior task-specific performance across the two datasets.

Taken together, the comparative experiments indicate task-dependent trade-offs rather than a single overall ordering across models. Some Transformer-based models remain competitive in one or both task-specific panels, whereas several CNN-based baselines remain comparatively stable in lower-complexity regions. Within this task-specific view, N-Unet offers a favorable efficiency profile together with competitive classification accuracy and strong segmentation performance, especially in Dice-based evaluation, without requiring these outcomes to be collapsed into a single aggregated score.

### 4.8. Analysis of Ablation Experiment Results

In the ablation study, we examine both the individual and combined contributions of the proposed components: AMTL, AFF, CLAE, and CSB. By incrementally integrating them into the standard Unet architecture, we assess their separate effects and their interactions in classification and segmentation.

As shown in Table 7, we designed a set of controlled experiments to evaluate the contribution of each component to breast ultrasound classification and segmentation. The evaluated modules are A (AMTL), a multi-task loss for balancing task learning; C (CSB), a conditional segmentation refinement mechanism based on classification outcomes; AF (AFF), an adaptive fusion strategy for richer contextual representation; and CL (CLAE), a decoder-side attention mechanism for semantic refinement.

Here, A denotes the complete AMTL design defined in Section 3.5, where the task-specific Focal-BCE and Dice objectives are used together with uncertainty-based weighting and task-number normalization as one integrated optimization scheme. Accordingly, Table 7 evaluates AMTL at the integrated loss-design level. We acknowledge that this ablation setting supports overall loss-level evaluation but does not isolate the individual contributions of the internal AMTL terms. Such finer-grained loss-component controls would provide stronger subcomponent-level attribution and remain future work.

Here, CL denotes the complete CLAE block as defined in Section 3.3, where CBAM-based channel–spatial recalibration and attention-gate filtering operate sequentially within one decoder-side refinement unit. Accordingly, Table 7 evaluates CLAE at the module level as a unified design. We acknowledge that this ablation setting supports integrated-module evaluation but does not isolate the individual contributions of CBAM and AG within CLAE. Such finer-grained controls would provide stronger evidence for subcomponent-level attribution and will be considered in future work.

#### 4.8.1. Impact of Individual Modules

We begin by examining the effect of adding each module individually to the baseline Unet model.

First, the A module (AMTL) yields a substantial improvement in both classification and segmentation. Classification accuracy rises from 0.8217 to 0.9167, and IOU increases from 0.4893 to 0.5252. These results indicate that AMTL balances the two optimization objectives effectively and improves coordination within the multi-task framework.

The CSB module exhibits more complex behavior when used alone. In that setting, it slightly reduces both classification accuracy and segmentation performance relative to the baseline. This occurs because CSB introduces a conditional masking mechanism that suppresses segmentation output for samples predicted as negative. If classification performance is not sufficiently strong, this suppression can incorrectly remove true positive regions. However, when CSB is combined with modules that strengthen classification, such as AMTL or AFF, performance improves substantially. This result confirms that CSB acts as a selective enhancer whose effectiveness depends on the reliability of the classification branch.

Next, the AF module provides noticeable gains in segmentation quality. By fusing multi-scale information across levels, it enriches contextual feature representation. When AFF is added alone, segmentation IOU increases to 0.5301 and classification accuracy also improves moderately. This result suggests that AFF strengthens the representational capacity of the model by reinforcing feature associations across scales.

Finally, the CL module yields the largest segmentation gain among the individual modules. IOU rises to 0.5439, and classification accuracy reaches 0.8896. This suggests that CLAE improves semantic consistency during decoding by refining upsampled features through attention, thereby enhancing both boundary detail and global feature understanding.

#### 4.8.2. Impact of Two-Module Combinations

We next analyze two-module combinations to examine their synergistic behavior.

The combination of A and C (AMTL + CSB) yields a marked performance boost, reaching a classification accuracy of 0.9154, an IOU of 0.6712, and a DC of 0.7318. This result illustrates the complementary nature of the two modules: AMTL stabilizes task balancing during training, whereas CSB enhances segmentation during inference through classification guidance. Together, they form a unified optimization-and-inference enhancement scheme.

The integration of A and AF (AMTL + AFF) also produces consistent gains. Classification accuracy reaches 0.8923, while IOU and DC rise to 0.5705 and 0.6358, respectively. In this setting, AFF enriches contextual feature diversity, and AMTL helps translate those richer representations into improvements in both tasks.

The pairing of A and CL (AMTL + CLAE) shows a particular advantage in segmentation. IOU and DC increase to 0.5859 and 0.6432, respectively, while classification accuracy reaches 0.9102. These results suggest that CLAE is especially effective when supported by the stable optimization behavior induced by AMTL, allowing the decoder to capture finer structural details and improve spatial consistency.

The combinations of C and AF (CSB + AFF) and C and CL (CSB + CLAE) further demonstrate the cooperative effect of CSB when paired with stronger feature extractors. For example, CSB + AFF achieves an accuracy of 0.8859, an IOU of 0.6635, and a DC of 0.7398. Similarly, CSB + CLAE raises segmentation performance to an IOU of 0.6459 and a DC of 0.7214, indicating that attention-driven refinement further strengthens CSB at inference time.

Finally, the AF + CL (AFF + CLAE) configuration also improves segmentation performance, reaching an IOU of 0.6011 and a DC of 0.6650. This result further supports the complementary roles of the two modules: AFF enriches multi-scale feature representation, whereas CLAE improves feature focus during decoding.

#### 4.8.3. Impact of Three-Module Combinations

We then analyze three-module combinations to evaluate deeper interactions among the proposed components.

The combination of A, C, and AF (AMTL + CSB + AFF) achieves the best performance among all tested three-module configurations, with a classification accuracy of 0.9497, an IOU of 0.7254, and a DC of 0.7798. This configuration combines task-level optimization, segmentation refinement, and contextual feature fusion effectively. The result suggests that it leverages both local and global cues while maintaining balanced optimization across tasks.

The combination of A, C, and CL (AMTL + CSB + CLAE) also yields excellent results, with a classification accuracy of 0.9436, an IOU of 0.7230, and a DC of 0.7939. These metrics highlight strong synergy between cross-level attention and task-specific guidance. CLAE improves feature consistency during decoding, while CSB refines segmentation according to the classification result under the balanced supervision of AMTL.

When A, AF, and CL (AMTL + AFF + CLAE) are integrated, the model achieves a classification accuracy of 0.9273 and segmentation scores of 0.6602 for IOU and 0.7101 for DC. This configuration enhances deep feature expressiveness through both fusion and attention. The presence of AMTL helps use those improved representations more effectively across tasks.

The configuration of C, AF, and CL (CSB + AFF + CLAE) achieves a classification accuracy of 0.9218 and segmentation scores of 0.6909 for IOU and 0.7583 for DC. This result again shows that AFF and CLAE improve feature quality and that CSB can exploit these improvements to produce more refined segmentation outputs.

Taken together, these results emphasize the complementary nature of the proposed modules within the multi-task framework. The interaction between optimization-driven, attention-driven, and inference-driven components improves both individual task performance and overall model capability.

#### 4.8.4. Impact of Full Module Integration

We conclude the ablation study by examining the effect of integrating all proposed modules into the network. The baseline Unet serves as the reference point, with a classification accuracy of 0.8217, a pixel accuracy of 0.9261, an IOU of 0.4893, and a DC of 0.5421.

As shown above, introducing individual modules such as A, C, AF, and CL improves different aspects of performance. AMTL mainly enhances classification, whereas CSB has a more pronounced influence on segmentation behavior. Two-module combinations, such as A + C and A + AF, produce broader gains across metrics, and three-module combinations, such as A + C + AF and A + C + CL, yield still stronger overall performance.

Ultimately, full integration of all four modules in N-Unet yields the best overall performance, with a classification accuracy of 0.9654, a pixel accuracy of 0.9675, an IOU of 0.7404, and a DC of 0.8070. This comprehensive improvement validates the effectiveness of the individual components and highlights their complementary roles when jointly applied.

#### 4.8.5. Comparison with Single-Task Counterparts

To directly quantify the overall benefit of joint learning beyond the module-level ablations in Table 7, we further compared the full N-Unet against a classification-only counterpart (N-Unet-Cls) and a segmentation-only counterpart (N-Unet-Seg) on the BUSI dataset, as summarized in Table 8. The multi-task model improves classification accuracy from 0.9407 to 0.9654 and F1-score from 0.9615 to 0.9728 relative to N-Unet-Cls. Relative to N-Unet-Seg, it further improves segmentation pixel accuracy from 0.9643 to 0.9675, IOU from 0.7198 to 0.7404, and Dice from 0.7847 to 0.8070. Although the recall of the multi-task model is slightly lower than that of N-Unet-Cls (0.9615 vs. 0.9642), its overall classification balance and segmentation quality are both improved. These results provide direct evidence that jointly learning classification and segmentation yields measurable overall benefits relative to training the two tasks independently.

### 4.9. Overall Summary

Overall, the comparative experiments show that N-Unet is the most balanced model across the two datasets. It achieves the highest accuracy in classification on both BUSI and BUS-UCLM while also delivering the best Dice coefficient and IOU in segmentation. Although Transformer-based models such as UNETR and SwinUnet attain higher recall in some classification settings, they do so at the cost of lower precision and more false-positive predictions. By contrast, N-Unet provides a better trade-off between sensitivity and specificity, together with consistently strong boundary delineation.

The ablation results further show that the performance gain does not arise from any single module alone. AMTL, AFF, CLAE, and CSB make complementary contributions, and the fully integrated model achieves the strongest overall performance, with a classification accuracy of 0.9654 and a segmentation DC of 0.8070. These findings indicate that coordinated multi-task optimization, Adaptive Feature Fusion, decoder attention refinement, and classification-guided segmentation consistency are all important to the final result.

## 5. Discussion

### 5.1. Overall Performance Summary

N-Unet achieves the highest classification accuracy and segmentation Dice on both the BUSI and BUS-UCLM benchmarks while maintaining the lowest confirmed parameter count and computational cost among all evaluated models. This combined outcome reflects a synergistic design rather than a task-level trade-off, demonstrating that improvements in both tasks can be realized simultaneously without sacrificing efficiency.

### 5.2. Component Interaction and Why the Method Works

The effectiveness of N-Unet can be understood by examining how its four components interact. **AMTL** replaces fixed scalar loss weights with learnable uncertainty parameters, preventing the gradient of one task from dominating training and enabling stable co-optimization across varying loss magnitudes. **AFF** performs adaptive multi-scale feature fusion across encoder levels, addressing the wide range of nodule sizes encountered in clinical data. **CLAE** introduces dual attention (channel and spatial) in the decoder, refining lesion boundary localization in regions with ambiguous tissue contrast. **CSB** imposes architectural consistency between the two branches: when a nodule is predicted (σ=0), the segmentation output passes through unchanged; when no nodule is predicted (σ=1), the output is suppressed to zero, ensuring both task outputs are semantically non-contradictory. Crucially, since all reported segmentation metrics are computed exclusively on nodule-containing images (Section 4.5.2), the CSB suppression mechanism does not inflate any reported segmentation score. CSB’s contribution is instead architectural—validated by the ablation result in Table 7, where AMTL and CSB jointly yield a substantially larger performance gain than either module applied alone. Taken together with the single-task comparison in Table 8, these results support the functional effectiveness of the proposed components within the present breast ultrasound setting, although they do not by themselves establish external generalization to ultrasound datasets from other anatomical regions.

### 5.3. Task-Specific Interpretation and Dataset Characteristics

Breast ultrasound analysis presents several intrinsic imaging challenges, as introduced in Section 1. Low image contrast and speckle noise—an inherent artifact of coherent ultrasound imaging—produce a granular background that obscures lesion boundaries. Iso-intensity, where benign and malignant tissue share visually similar appearances despite different cellular origins, and hypointensity, where certain lesions appear as darker regions with reduced imaging signal, both complicate reliable image-level classification. Individual patient anatomy, suboptimal acquisition conditions, and variations in tumor size further raise inter-sample variability and degrade generalization. Finally, breast nodule sizes span nearly two orders of magnitude with frequent irregular morphology, creating a highly heterogeneous segmentation target. The N-Unet components directly address these challenges: AFF provides adaptive cross-level feature fusion to handle multi-scale size variability; CLAE refines boundary delineation in low-contrast and iso-intense tissue regions via dual channel–spatial attention; and AMTL stabilizes joint optimization under the task-gradient imbalance that arises from coupling the classification and segmentation losses. CSB further addresses a clinically relevant consistency requirement in BUS analysis by suppressing semantically implausible foreground predictions on images classified as normal. Together, these correspondences clarify the breast ultrasound-oriented design rationale of N-Unet in the present study, although they should not be interpreted as direct evidence of transferability to other ultrasound domains.

The performance differential between BUSI and BUS-UCLM (Dice: 80.70% vs. 92.16%) is attributable to intrinsic distributional differences between the two datasets. First, BUS-UCLM contains 264 lesion-containing samples (174 benign + 90 malignant, Section 4.1) compared with 647 in BUSI (437 benign + 210 malignant). This smaller pool of unique lesion presentations concentrates the segmentation learning signal around a narrower range of lesion appearances, reducing the effective diversity of the generalization demand. Second, the nodule size stratification thresholds derived from the two datasets (BUS-UCLM: α=1811, β=4672 pixels; BUSI: α=2097, β=6725 pixels, at 256×256 resolution) indicate that BUS-UCLM lesions span a narrower and more compact size range (interquartile spread: 2861 vs. 4628 pixels), contributing to a more consistent and tractable segmentation training target. The same direction of performance differential is observed across all compared models, confirming that this gap reflects a dataset-level property rather than a model-specific advantage.

### 5.4. Failure Case Behavior and CSB Risk Asymmetry

A case-specific analysis of four representative failure modes is provided in Section 4.6.3 and Figure 11. These failures belong to two distinct categories. The first category comprises classification-induced failures directly mediated by CSB: case (a) shows a false-positive classification (σ=0), leaving the CSB gate fully open, whereupon the segmentation sub-network independently produces a spurious activation; case (b) shows a false-negative classification (σ=1), deterministically suppressing the entire segmentation output via the hard binary gate. The second category comprises pure segmentation failures that are unrelated to the CSB mechanism: case (c) shows the segmentation sub-network misled by a distracting non-lesion structure, and case (d) shows incomplete delineation of a large or morphologically irregular lesion—both arising from intrinsic limitations of the segmentation sub-network rather than from CSB gating. For the classification-induced category, the CSB formulation admits an analytically tractable asymmetric risk analysis. Under $\hat{S}=\left(1-\sigma \right)\cdot S$, a false-negative error (σ=1) zeros the segmentation output deterministically, regardless of the segmentation sub-network’s internal state, whereas a false-positive error (σ=0) leaves the gate open and passes the segmentation output through unmodified; whether any spurious activation appears then depends entirely on the independent output of the segmentation sub-network on that image, making this a conditional rather than deterministic failure. The gate is strictly unidirectional—segmentation output does not influence the classification decision—ruling out circular dependency. This asymmetry implies that false-negative errors carry a higher architectural risk and motivates future exploration of soft, confidence-weighted gating strategies.

### 5.5. Limitations

Several limitations of the current study are acknowledged. First, both datasets are single-institution 2D collections; cross-center generalizability under diverse imaging protocols, devices, and patient demographics has not been directly validated. Although five-fold cross-validation helps reduce variance in the performance estimate, it does not eliminate the risk of overfitting on relatively limited public datasets. In addition, the current evaluation remains confined to breast ultrasound and does not include external testing on ultrasound datasets from other anatomical regions. This constraint also reflects an objective practical barrier: the collection and annotation of large-scale medical imaging datasets requires specialized acquisition equipment, expert radiologist involvement, and ethics approval for patient data, making comprehensive multi-center data collection resource-intensive and time-consuming in practice. Second, the hard binary gate in CSB creates a deterministic segmentation failure for every false-negative classification error (Figure 11, case (b)), an intrinsic risk of the current formulation. Third, data augmentation relies exclusively on standard geometric transformations; ultrasound-specific strategies such as speckle noise injection, contrast perturbation, and tissue deformation were not employed, which may limit robustness under variable clinical acquisition conditions. This limitation should be kept in mind when interpreting the reported robustness of the current model under broader clinical imaging variability. Fourth, although lesion-size stratification was used during fold construction, the present study does not report a formal size-wise breakdown of segmentation metrics across small, medium, and large nodules, so lesion-scale-specific strengths and failure modes are not yet quantified. Fifth, the architecture operates on individual 2D frames and does not leverage the volumetric information available in 3D ultrasound acquisitions. Sixth, inter-rater annotation variability was not modeled, as both datasets provide only a single reference mask per image.

### 5.6. Future Work

Several directions for future research are identified. A soft or confidence-weighted variant of CSB—replacing the binary gate with a continuous score derived from classification confidence—would mitigate deterministic suppression under uncertain predictions. Ultrasound-specific augmentation strategies, including speckle simulation, local contrast variation, and artifact synthesis, would improve domain robustness under variable imaging conditions. A more systematic investigation of BUS-specific augmentation strategies would be valuable in future work. Multi-center validation across heterogeneous devices and acquisition protocols, together with evaluation on ultrasound datasets from other anatomical regions, would more rigorously assess external generalization. Extending the framework to 3D volumetric ultrasound or temporal sequences represents a longer-term direction for enhanced clinical applicability. In addition, dedicated experiments that vary training-set size and evaluate alternative Transformer-specific training protocols would help clarify whether the lower segmentation performance of Transformer-based baselines such as UNETR and SwinUnet is more closely related to data regime, training protocol, or architectural inductive bias. A dedicated size-wise segmentation analysis under the small-, medium-, and large-nodule definitions of Section 4.1 would also be valuable for clarifying which lesion scales remain most challenging. Broader comparison with additional recent Transformer-based baselines under the same joint classification-and-segmentation protocol would also be valuable in future work. Incorporating multi-rater annotations or uncertainty-aware evaluation would further address annotation variability.

## 6. Conclusions

In this study, we proposed N-Unet, a multi-task deep learning model for breast ultrasound image classification and segmentation. By integrating AMTL, AFF, CLAE, and CSB within a unified framework, N-Unet achieves strong performance on both BUSI and BUS-UCLM. The model attains the highest classification accuracy on both datasets and the best segmentation Dice and IOU, while maintaining a favorable balance between sensitivity and specificity.

These results indicate that coordinated multi-task optimization and task-aware feature interaction are effective for breast ultrasound analysis. N-Unet is also computationally efficient, requiring only 8.95 M parameters and 14.74 GFLOPs, which supports its practical deployment potential.

Several limitations remain. The current evaluation is still restricted to relatively limited public datasets, and broader multi-institutional validation is needed to assess generalizability more rigorously. In addition, Transformer-based models achieve very high recall but also produce more false positives, suggesting that the trade-off between global context modeling and localized precision deserves further study. Future work should therefore focus on larger and more diverse datasets, improved multi-task interaction mechanisms, and possible integration with multi-modal, self-supervised, or domain-adaptive learning strategies.

## Figures and Tables

**Table 1:** Comparison between standard convolution and depthwise separable (DS) convolution.

Aspect	Standard Convolution	DS Convolution
Operation	Full K×K on all channels	Depthwise + pointwise
Parameters	K×K×M×N	K×K×M+1×1×M×N
Computation	High	Low
Efficiency	Large, slow	Small, fast
Feature learning	Spatial + channel jointly	Spatial + channel separately

**Table 2:** Performance comparison of different models for the classification task on the BUSI dataset (Mean ± SD).

Model	Precision	Recall	F1-Score	Accuracy
Unet [22]	0.8658 ± 0.0124	0.9556 ± 0.0085	0.9085 ± 0.0102	0.8344 ± 0.0156
M-Unet [43]	0.9762 ± 0.0054	0.9462 ± 0.0091	0.9609 ± 0.0068	0.9363 ± 0.0074
ResUnet [44]	0.9197 ± 0.0112	0.9767 ± 0.0063	0.9474 ± 0.0089	0.9108 ± 0.0105
AGUnet [11]	0.9618 ± 0.0072	0.9767 ± 0.0051	0.9692 ± 0.0061	0.9490 ± 0.0065
ANUnet [23]	0.9764 ± 0.0048	0.9538 ± 0.0077	0.9650 ± 0.0059	0.9427 ± 0.0062
BTS-Unet [28]	0.9694 ± 0.0081	0.9500 ± 0.0088	0.9596 ± 0.0083	0.9333 ± 0.0091
UNETR [25]	0.9078 ± 0.0153	**0.9922** ± 0.0034	0.9481 ± 0.0094	0.9067 ± 0.0128
SwinUnet [26]	0.8909 ± 0.0141	0.9899 ± 0.0042	0.9378 ± 0.0098	0.8917 ± 0.0113
MTF-Unet [27]	0.9343 ± 0.0096	0.9846 ± 0.0055	0.9588 ± 0.0071	0.9299 ± 0.0084
ACSNet [29]	0.9500 ± 0.0087	0.9596 ± 0.0079	0.9548 ± 0.0082	0.9250 ± 0.0088
N-Unet (Ours)	**0.9843** ± 0.0041	0.9615 ± 0.0064	**0.9728** ± 0.0050	**0.9654** ± 0.0049

Bold indicates the best-performing value in each column.

**Table 3:** Performance comparison of different models for the classification task on the BUS-UCLM dataset (Mean ± SD).

Model	Precision	Recall	F1-Score	Accuracy
Unet [22]	0.7879 ± 0.0185	0.6500 ± 0.0285	0.7123 ± 0.0212	0.7900 ± 0.0194
M-Unet [43]	0.7931 ± 0.0164	0.6216 ± 0.0242	0.6970 ± 0.0188	0.7917 ± 0.0156
ResUnet [44]	0.8125 ± 0.0152	0.7027 ± 0.0198	0.7536 ± 0.0164	0.8229 ± 0.0142
AGUnet [11]	0.8333 ± 0.0148	0.6944 ± 0.0215	0.7576 ± 0.0159	0.8333 ± 0.0135
ANUnet [23]	0.9697 ± 0.0072	0.8421 ± 0.0142	0.9014 ± 0.0098	0.9300 ± 0.0084
BTS-Unet [28]	0.9688 ± 0.0078	0.8158 ± 0.0156	0.8857 ± 0.0112	0.9200 ± 0.0091
UNETR [25]	0.9074 ± 0.0124	**0.9899** ± 0.0038	**0.9469** ± 0.0074	0.9083 ± 0.0108
SwinUnet [26]	0.9074 ± 0.0119	0.9800 ± 0.0042	0.9423 ± 0.0082	0.9000 ± 0.0115
MTF-Unet [27]	0.9630 ± 0.0086	0.7027 ± 0.0224	0.8125 ± 0.0148	0.8800 ± 0.0122
ACSNet [29]	**1.0000** ± 0.0000	0.8286 ± 0.0135	0.9062 ± 0.0104	0.9400 ± 0.0081
N-Unet (Ours)	0.9667 ± 0.0082	0.9062 ± 0.0114	0.9355 ± 0.0089	**0.9583** ± 0.0048

Bold indicates the best-performing value in each column.

**Table 4:** Performance comparison of different models for the segmentation task on the BUSI dataset (Mean ± SD).

Model	SE	PC	DC	IOU	Acc
Unet [22]	0.6137 ± 0.0452	0.5937 ± 0.0418	0.5421 ± 0.0487	0.4893 ± 0.0496	0.9261 ± 0.0125
M-Unet [43]	0.7842 ± 0.0215	0.7631 ± 0.0234	0.7510 ± 0.0248	0.6827 ± 0.0312	0.9572 ± 0.0084
ResUnet [44]	0.6645 ± 0.0385	0.6358 ± 0.0354	0.6234 ± 0.0398	0.5569 ± 0.0415	0.9423 ± 0.0102
AGUnet [11]	0.6472 ± 0.0364	0.6521 ± 0.0321	0.6064 ± 0.0372	0.5227 ± 0.0394	0.9482 ± 0.0095
ANUnet [23]	0.7853 ± 0.0198	0.8152 ± 0.0165	0.7854 ± 0.0184	0.7226 ± 0.0215	0.9617 ± 0.0072
BTS-Unet [28]	0.7153 ± 0.0284	0.6763 ± 0.0292	0.6767 ± 0.0275	0.6042 ± 0.0324	0.9441 ± 0.0098
UNETR [25]	0.7032 ± 0.0256	0.6427 ± 0.0315	0.6998 ± 0.0242	0.6185 ± 0.0284	0.9554 ± 0.0089
SwinUnet [26]	0.7473 ± 0.0224	0.7265 ± 0.0218	0.7208 ± 0.0235	0.6531 ± 0.0267	0.9703 ± 0.0065
MTF-Unet [27]	0.7895 ± 0.0182	0.8119 ± 0.0154	0.7838 ± 0.0176	0.7166 ± 0.0208	**0.9736** ± 0.0051
ACSNet [29]	0.7937 ± 0.0175	0.8004 ± 0.0168	0.7846 ± 0.0182	0.7201 ± 0.0195	0.9674 ± 0.0068
N-Unet (Ours)	**0.8114** ± 0.0125	**0.8400** ± 0.0112	**0.8070** ± 0.0134	**0.7404** ± 0.0158	0.9675 ± 0.0062

Bold indicates the best-performing value in each column.

**Table 5:** Performance comparison of different models for the segmentation task on the BUS-UCLM dataset (Mean ± SD).

Model	SE	PC	DC	IOU	Acc
Unet [22]	0.6705 ± 0.0342	0.6401 ± 0.0318	0.6417 ± 0.0365	0.6065 ± 0.0384	0.9784 ± 0.0058
M-Unet [43]	0.6895 ± 0.0315	0.6923 ± 0.0284	0.6716 ± 0.0302	0.6332 ± 0.0335	0.9727 ± 0.0064
ResUnet [44]	0.7950 ± 0.0248	0.8126 ± 0.0215	0.7849 ± 0.0234	0.7505 ± 0.0268	0.9852 ± 0.0042
AGUnet [11]	0.8199 ± 0.0205	0.8177 ± 0.0198	0.8143 ± 0.0182	0.7879 ± 0.0215	0.9865 ± 0.0039
ANUnet [23]	0.7848 ± 0.0264	0.7591 ± 0.0287	0.7654 ± 0.0251	0.7372 ± 0.0294	0.9781 ± 0.0052
BTS-Unet [28]	0.7897 ± 0.0252	0.7974 ± 0.0224	0.7712 ± 0.0241	0.7391 ± 0.0275	0.9868 ± 0.0038
UNETR [25]	0.7532 ± 0.0298	0.7398 ± 0.0312	0.7283 ± 0.0289	0.6985 ± 0.0324	0.9766 ± 0.0061
SwinUnet [26]	0.7602 ± 0.0275	0.7803 ± 0.0241	0.7564 ± 0.0262	0.6879 ± 0.0308	0.9692 ± 0.0075
MTF-Unet [27]	0.8582 ± 0.0164	0.8702 ± 0.0142	0.8547 ± 0.0158	0.8243 ± 0.0184	0.9851 ± 0.0045
ACSNet [29]	0.9239 ± 0.0115	0.9081 ± 0.0124	0.9115 ± 0.0108	0.8857 ± 0.0142	0.9913 ± 0.0028
N-Unet (Ours)	**0.9329** ± 0.0098	**0.9215** ± 0.0105	**0.9216** ± 0.0092	**0.8974** ± 0.0124	**0.9917** ± 0.0025

Bold indicates the best-performing value in each column.

**Table 6:** Complexity comparison of models with confirmed parameters and FLOPs statistics.

Model	Params (M)	FLOPs (G)
N-Unet (Ours)	**8.95**	**14.74**
M-Unet [43]	18.50	16.40
SwinUnet [26]	86.09	18.22
ANUnet [23]	24.60	21.70
Unet [22]	29.38	25.69
AGUnet [11]	31.20	27.40
ACSNet [29]	42.99	30.96
ResUnet [44]	40.23	29.88
MTF-Unet [27]	27.31	109.12
UNETR [25]	113.12	113.36
BTS-Unet [28]	25.02	142.87

Bold indicates the lowest value in each column.

**Table 7:** Ablation study of different Unet-based models with various modules for classification and segmentation tasks (Mean ± SD).

Model Name	AMTL	CSB	AFF	CLAE	Classification		Segmentation		
					Acc		Pixel Acc	IOU	DC
Unet					0.8217 ± 0.0165		0.9261 ± 0.0125	0.4893 ± 0.0496	0.5421 ± 0.0487
AMTLUnet	✓				0.9167 ± 0.0112		0.9173 ± 0.0134	0.5252 ± 0.0415	0.6056 ± 0.0392
CSBUnet		✓			0.8185 ± 0.0172		0.8943 ± 0.0158	0.4799 ± 0.0512	0.5374 ± 0.0504
AFFUnet			✓		0.8723 ± 0.0138		0.9356 ± 0.0118	0.5301 ± 0.0428	0.5927 ± 0.0385
CLAEUnet				✓	0.8896 ± 0.0125		0.9332 ± 0.0121	0.5439 ± 0.0406	0.6088 ± 0.0374
A_CUnet	✓	✓			0.9154 ± 0.0104		0.9568 ± 0.0092	0.6712 ± 0.0284	0.7318 ± 0.0245
A_AFFUnet	✓		✓		0.8923 ± 0.0118		0.9384 ± 0.0112	0.5705 ± 0.0356	0.6358 ± 0.0312
A_CLAEUnet	✓			✓	0.9102 ± 0.0109		0.9396 ± 0.0108	0.5859 ± 0.0342	0.6432 ± 0.0305
CSB_AFFUnet		✓	✓		0.8859 ± 0.0122		0.9484 ± 0.0105	0.6635 ± 0.0298	0.7398 ± 0.0264
CSB_CLAEUnet		✓		✓	0.8832 ± 0.0128		0.9403 ± 0.0114	0.6459 ± 0.0315	0.7214 ± 0.0278
AFF_CLAEUnet			✓	✓	0.8904 ± 0.0121		0.9317 ± 0.0119	0.6011 ± 0.0334	0.6650 ± 0.0298
A_CSB_AFFUnet	✓	✓	✓		0.9497 ± 0.0075		0.9612 ± 0.0084	0.7254 ± 0.0215	0.7798 ± 0.0182
A_CSB_CLAEUnet	✓	✓		✓	0.9436 ± 0.0078		0.9581 ± 0.0088	0.7230 ± 0.0221	0.7939 ± 0.0175
A_AFF_CLAEUnet	✓		✓	✓	0.9273 ± 0.0092		0.9378 ± 0.0104	0.6602 ± 0.0284	0.7101 ± 0.0256
CSB_AFF_CLAEUnet		✓	✓	✓	0.9218 ± 0.0098		0.9505 ± 0.0091	0.6909 ± 0.0242	0.7583 ± 0.0218
N-Unet (Ours)	✓	✓	✓	✓	**0.9654** ± 0.0049		**0.9675** ± 0.0062	**0.7404** ± 0.0158	**0.8070** ± 0.0134

Bold indicates the best-performing value in each metric column. ✓ indicates that the corresponding module is included in the model configuration.

**Table 8:** Comparison between single-task and multi-task variants of N-Unet on the BUSI dataset (Mean ± SD).

Model	Precision	Recall	F1-Score	Accuracy	Pixel Acc	IOU	DC
N-Unet-Cls	0.9589 ± 0.0084	0.9642 ± 0.0076	0.9615 ± 0.0079	0.9407 ± 0.0091	-	-	-
N-Unet-Seg	-	-	-	-	0.9643 ± 0.0082	0.7198 ± 0.0215	0.7847 ± 0.0194
N-Unet (Ours)	**0.9843** ± 0.0041	0.9615 ± 0.0064	**0.9728** ± 0.0050	**0.9654** ± 0.0049	**0.9675** ± 0.0062	**0.7404** ± 0.0158	**0.8070** ± 0.0134

Bold indicates the best-performing value in each metric column.

## Data Availability

The original data presented in the study are openly available in the BUSI dataset at https://scholar.cu.edu.eg/?q=afahmy/pages/dataset (accessed on 5 March 2026), and in the BUS-UCLM dataset at https://data.mendeley.com/datasets/7fvgj4jsp7/1 (accessed on 5 March 2026). Some or all data, models, or codes generated or used during the study are available from the corresponding author upon reasonable request due to privacy considerations.

## Abbreviations

The following abbreviations are used in this manuscript:

BUS	Breast ultrasound
BUSI	Breast Ultrasound Image Dataset
MTL	Multi-task learning
AFF	Adaptive Feature Fusion
CLAE	Cross-Level Attention Enhancement
CSB	Conditional Segmentation Boosting
AMTL	Adaptive Multi-Task Loss
GT	Ground truth
IOU	Intersection over Union
DC	Dice coefficient
